# Disseminated intravascular coagulation phenotype is regulated by the TRPM7 channel during sepsis

**DOI:** 10.1186/s40659-023-00419-4

**Published:** 2023-03-03

**Authors:** Ivanka Jiménez-Dinamarca, Yolanda Prado, Pablo Tapia, Sebastian Gatica, Clemens Alt, Charles P. Lin, Cristian Reyes-Martínez, Carmen G. Feijóo, Cristobal Aravena, Alejandra González-Canacer, Simón Correa, Diego Varela, Claudio Cabello-Verrugio, Felipe Simon

**Affiliations:** 1 Laboratory of Integrative Physiopathology, Faculty of Life Sciences, Universidad Andres Bello, Republica 330, 8370186 Santiago, Chile; 2 Millennium Institute On Immunology and Immunotherapy, Santiago, Chile; 3 Unidad de Paciente Crítico Adulto, Hospital Clínico La Florida, Santiago, Chile; 4 Center for Systems Biology and Wellman Center for Photomedicine, Massachusetts General Hospital and Harvard Medical School, Boston, MA USA; 5 Fish Immunology Laboratory, Faculty of Life Sciences, Universidad Andres Bello, Santiago, Chile; 6 Programa de Fisiología Y Biofísica, Facultad de Medicina, Instituto de Ciencias Biomédicas, Universidad de Chile, Santiago, Chile; 7 Millennium Nucleus of Ion Channel-Associated Diseases, Santiago, Chile; 8 Laboratory of Muscle Pathology, Fragility and Aging, Faculty of Life Sciences, Universidad Andres Bello, Republica 330, 8370186 Santiago, Chile; 9 Center for the Development of Nanoscience and Nanotechnology (CEDENNA), Universidad de Santiago de Chile, Santiago, Chile

**Keywords:** TRPM7, Coagulation, Sepsis, Endothelium, Organ dysfunction

## Abstract

**Background:**

Sepsis is an uncontrolled inflammatory response against a systemic infection that results in elevated mortality, mainly induced by bacterial products known as endotoxins, producing endotoxemia. Disseminated intravascular coagulation (DIC) is frequently observed in septic patients and is associated with organ failure and death. Sepsis activates endothelial cells (ECs), promoting a prothrombotic phenotype contributing to DIC. Ion channel-mediated calcium permeability participates in coagulation. The transient reception potential melastatin 7 (TRPM7) non-selective divalent cation channel that also contains an α-kinase domain, which is permeable to divalent cations including Ca^2+^, regulates endotoxin-stimulated calcium permeability in ECs and is associated with increased mortality in septic patients. However, whether endothelial TRPM7 mediates endotoxemia-induced coagulation is not known. Therefore, our aim was to examine if TRPM7 mediates coagulation during endotoxemia.

**Results:**

The results showed that TRPM7 regulated endotoxin-induced platelet and neutrophil adhesion to ECs, dependent on the TRPM7 ion channel activity and by the α-kinase function. Endotoxic animals showed that TRPM7 mediated neutrophil rolling on blood vessels and intravascular coagulation. TRPM7 mediated the increased expression of the adhesion proteins, von Willebrand factor (vWF), intercellular adhesion molecule 1 (ICAM-1), and P-selectin, which were also mediated by the TRPM7 α-kinase function. Notably, endotoxin-induced expression of vWF, ICAM-1 and P-selectin were required for endotoxin-induced platelet and neutrophil adhesion to ECs. Endotoxemic rats showed increased endothelial TRPM7 expression associated with a procoagulant phenotype, liver and kidney dysfunction, increased death events and an increased relative risk of death. Interestingly, circulating ECs (CECs) from septic shock patients (SSPs) showed increased TRPM7 expression associated with increased DIC scores and decreased survival times. Additionally, SSPs with a high expression of TRPM7 in CECs showed increased mortality and relative risk of death. Notably, CECs from SSPs showed significant results from the AUROC analyses for predicting mortality in SSPs that were better than the Acute Physiology and Chronic Health Evaluation II (APACHE II) and the Sequential Organ Failure Assessment (SOFA) scores.

**Conclusions:**

Our study demonstrates that sepsis-induced DIC is mediated by TRPM7 in ECs. TRPM7 ion channel activity and α-kinase function are required by DIC-mediated sepsis-induced organ dysfunction and its expression are associated with increased mortality during sepsis. TRPM7 appears as a new prognostic biomarker to predict mortality associated to DIC in SSPs, and as a novel target for drug development against DIC during infectious inflammatory diseases.

**Supplementary Information:**

The online version contains supplementary material available at 10.1186/s40659-023-00419-4.

## Introduction

Sepsis syndrome is a life-threatening and dysregulated inflammatory host response to infectious processes [1]. This condition strongly disturbs physiological and biochemical balances [2, 3] and results in an elevated mortality rate and high economic burden, positioning this syndrome as a public health challenge despite being extensively studied [1, 4].

Sepsis syndrome is mainly induced by toxic bacterial products known as endotoxins, such as lipopolysaccharide (LPS), that circulates into the bloodstream producing endotoxemia [5]. During the clinical progression of sepsis syndrome, several critical pathological dysfunctions are observed, including altered blood pressure, loss of temperature control, and coagulation disorders that usually result in disseminated intravascular coagulation (DIC) [6, 7]. DIC is an acquired syndrome of unlocalized vascular coagulation activation promoting organ dysfunction [8]. Importantly, coagulation system dysfunction is directly associated with the poor prognosis of septic patients and is associated with widespread vascular thrombus formation and underlying organ failure and death [9, 10].

Endothelial cells (ECs) located in the luminal face of blood vessels play a critical role in the onset, progression and diagnosis of DIC [8, 11]. Sepsis promotes EC activation and shifts them from an antiadhesive and antithrombotic phenotype into a proadhesive and prothrombotic state [11]. ECs exposed to an endotoxin challenge become activated and promote broad cell adhesion, such as platelet and neutrophil adhesion [12]

Endotoxic environments also promote platelet and neutrophil activation and their subsequent interaction [13]. This interplay, designated as platelet-neutrophil aggregates (PNAs), is a key trigger of DIC [14]. The adhesion of platelets and neutrophils on ECs surface is mediated by adhesion protein expression and translocation to the cell surface [15, 16]. Several adhesion proteins in activated ECs participate in platelet and neutrophil adhesion, including von Willebrand factor (vWF) intercellular adhesion molecule-1 (ICAM-1) and P-selectin [17]. It has been broadly reported by our group and others that P-selectin, ICAM-1 and vWF expression is induced by the nuclear factor-κB (NF-κB) transcription factor in activated ECs induced by endotoxin [18, 19], leading to platelet and neutrophil adhesion to ECs.

Interestingly, calcium signaling is a hallmark of coagulation and has a remarkable role as a second messenger in ECs [20, 21] and regulates the expression of adhesion molecules such as ICAM-1 [22]. In addition, it is known that inhibition of ion channels involved in calcium permeability decreases coagulation [23]. Of note, the use of calcium channel blockers has been shown to reduce mortality in endotoxemic rats and septic patients [24–26].

Considering its therapeutic implications, identifying the calcium channel that mediates calcium permeability to promote coagulation is an important issue. It has been reported that the transient reception potential melastatin 7 (TRPM7) non-selective cation channel, which is permeable to divalent cations including Ca^2+^, is a key regulator of calcium permeability in ECs under conditions of endotoxin stimulation, promoting several deleterious actions, including cytokine production, endothelial fibrosis, kidney failure and mortality [27, 28]. TRPM7 was discovered by several groups [29–31] and contains a cytosolic α-kinase domain [31, 32], which participates in several function including T cell activity, store operated calcium entry (SOCE) modulation, and arterial thrombosis [33–35].

Furthermore, TRPM7 is associated with increased mortality in septic patients and other septic models [36–38]. These findings suggest that TRPM7 could promote an EC-mediated coagulation phenotype upon septic challenge. However, the role of TRPM7 in the endotoxin-induced coagulation phenotype is currently not known. Therefore, the aim of this study was to examine if the TRPM7 channel mediates coagulation during endotoxemia.

The results presented here show that TRPM7 is required for endotoxin-induced platelet, neutrophil and platelet-neutrophil aggregate adhesion to ECs. In vivo experiments in endotoxic animals showed that TRPM7 mediates neutrophil rolling on blood vessels and intravascular coagulation. The underlying mechanism indicates TRPM7-mediated increased expression of the adhesion proteins vWF, ICAM-1 and P-selectin and NF-κB signaling. Endotoxemic rats showed that TRPM7 promotes a procoagulant phenotype and increases death and the relative risk of death, since TRPM7 downregulation protects against those endotoxemia-mediated features. Of note, TRPM7 expression was shown to be enhanced in the endothelium from endotoxemic rats and to be associated with survival time. Furthermore, increased TRPM7 expression was shown to be associated with liver and kidney failure as a mechanism for endotoxemia-mediated death. Interestingly, circulating mature endothelial cells (CMECs) and circulating endothelial progenitor cells (CEPCs) from septic shock patients (SSPs) showed increased TRPM7 expression, which was associated with increased DIC scores and decreased survival times. Additionally, SSPs with a high expression of TRPM7 in CMECs and CEPCs showed increased mortality and relative risk of death. Notably, CMECs and CEPCs from SSPs showed significant results from the AUROC analyses for predicting mortality in SSPs, and both parameters were better for such a prediction than APACHE II and SOFA scores.

Taken together, the prothrombotic phenotype induced by endotoxemia requires TRPM7 activity, which promotes DIC-mediated organ dysfunction and death during sepsis. Therefore, TRPM7 is a new target for treatment to decrease DIC during infectious inflammatory diseases and a new biomarker to predict mortality in SSPs.

## Materials and methods

### Cell culture

Human umbilical vein endothelial cell (HUVEC)-derived endothelial cell line EA.hy926 was cultured in Dulbecco’s Modified Eagle Medium Low (DMEM-Low) with 10% fetal bovine serum (FBS), 4 mM l-glutamine, 1 g/L d-glucose, 100 μg/mL penicillin/streptomycin (Pen/Strep) and 2.5 μg/mL amphotericin. Cells were grown in an incubator at 37 °C in a humidified atmosphere (95% air and 5% CO_2_).

### Platelets to endothelial cells adhesion assay

Endothelial cells were plated at a density of 2.5 × 10^4^ cells in a 96-well plate, and after 24 h were pretreated 1 h before and during the experiment with the non-specific pharmacological TRPM7 blockers, 2-Aminoethoxydiphenyl borate (2-APB) (100 µM (Sigma, USA)) and Carvacrol (0.732 M, (Sigma, USA)), the TRPM7 α-kinase domain inhibitor TG100-115 (1 µM (STCB, USA)), the vWF inhibitor Caplacizumab (0.8 µg/mL, (MCE, USA)), the ICAM-1 inhibitor A-286982 and A-205804 (25 nM and 25 nM, respectively (STCB, USA)), or the P-Sel inhibitor KF38789 (50 nM (STCB, USA)), or transfected with an siRNA against TRPM7 (siRNA^*TRPM7*^) or a non-targeting siRNA (siRNA^*Nontarget*^). In brief, cells were plated overnight in 96-well or 6-well plates and transfected with 5 nmol/L siRNA (Dharmacon) using lipofectamine (Invitrogen) and Opti-MEM (Gibco) according to manufacturer instructions for 4 h. Experiments were performed 48 h after transfection. To start the assay, LPS (O127:B8, Sigma-Aldrich) was added at a final concentration of 20 µg/mL with fluorescent-labeled platelets (~ 2.25 × 10^6^ platelets per well, contained in 100 µL of minimal experimentation medium) for 24 h at 37 ℃. Platelets were stained with the green fluorescent dye vibrant DiO (ThermoFisher Scientific) for 15 min at 37 °C. Then, non-adherent platelets were washed thrice with warm phosphate-buffered saline (PBS) and observed in the FLoid Cell Imaging Station (ThermoFisher Scientific). This experiment was performed in technical triplicate, and six fields for each condition were analyzed.

### Platelet isolation from human peripheral blood

The whole blood samples were obtained from normal volunteer donors into a Vacutainer tube with sodium citrate as anticoagulant. For each experiment 12 mL was collected. The extracted blood was transferred to 15 mL conical tubes and centrifuged at 200·g for 30 min at room temperature (RT). Once centrifuged, the upper portion of the plasma, corresponding to platelet-rich plasma (PRP), was transferred to a new tube, and HEPES Buffer (140 mM NaCl, 2.7 mM KCl, 3.8 mM HEPES, 5 mM EGTA, pH 7.4) was added in a 1:1 ratio, mixing it by inversion three times. Subsequently, the samples were centrifuged at 400·g for 30 min at RT to recover the supernatant again. To the latter, acid-citrate-dextrose (ACD) buffer (39 mM citric acid, 75 mM sodium citrate, 135 mM dextrose, pH 7.4) was added in 1:10 proportion, and then was mixed by inversion three times, centrifuged for 15 min at 3000·g at RT, and the pellet was recovered and resuspended in HEPES-Tyrode’s buffer free of MgCl_2_. Subsequently, the isolated platelets were counted using a Neubauer chamber and suspended at a concentration of ~ 2.25 × 10^6^ platelets/mL and stained with the green fluorescent dye vibrant DiO (ThermoFisher Scientific) for 15 min at 37 °C. Platelets were washed by centrifugation at 3000·g for 5 min at RT three times. The pellet was resuspended in DMEM-Low 1% FBS and 100 µL of medium containing ~ 2.25 × 10^6^ platelets was added to the endothelial cells' co-culture.

### Neutrophils to endothelial cells adhesion assay

Endothelial cells were plated at a density of ~ 2.5 × 10^4^ cells in a 96-well plate, and after 24 h were pretreated with calcium channels blockers or transfected with siRNA^*TRPM7*^ or siRNA^*Nontarget*^. To start the assay, LPS (O127:B8, Sigma-Aldrich) was added at a final concentration of 20 µg/mL for 24 h at 37 °C and then, pre-isolated and stained neutrophils (~ 2.5 × 10^4^ per well contained in 100 µL of minimal experimentation medium) were added for 30 min. Neutrophils were stained with the red fluorescent dye vibrant DiD (ThermoFisher Scientific) for 10 min at 37 °C. Finally, unattached neutrophils were removed by three careful washes with PBS. The attached neutrophils were determined by fluorescence microscopy and the analysis was performed as same as platelets.

### Neutrophils isolation from human peripheral blood

The isolation of neutrophils was performed with MACSxpress Neutrophil Isolation Kit and MACSxpress Separator (MASCS Miltenyi Biotec) as manufacturer’s instructions. In brief, the whole blood is obtained from normal volunteer donors with larger butterflies into a Vacutainer tube with EDTA as an anticoagulant. For each experiment 4 mL are collected and incubated with 2 mL pre-prepared MACSxpress Neutrophil Isolation Cocktail for 5 min. Then the tube is placed in the magnetic field of the MACSxpress Separator for 20 min. Then, the supernatant was collected and transferred into a new tube. Isolated neutrophils are stained with the red fluorescent dye vibrant DiD (ThermoFisher Scientific) for 10 min at 37 °C. Dye excess was washed by centrifugation at 1500 RPM for 5 min at room temperature after which the pellet was resuspended in DMEM-Low Serum-Free Medium (SFM). Washed neutrophils were counted using the Automated Cell Counter LUNA (Logos Biosystem) and diluted in DMEM-Low 1% FBS and then, 100 µL of medium containing ~ 2.5 × 10^4^ neutrophils was added to the endothelial cells' co-culture.

### Quantification and analysis of platelet and neutrophil adhesion to endothelial cells

The images obtained were processed in grayscale using a pixel analysis software designed for these experiments. Briefly, the mean size of a platelet or a neutrophil unit was calculated in pixels. Then, the number of total platelets or total neutrophils per image was calculated as the fluorescence intensity of each pixel, then were grouped into 5 grays scales to apply the intensity correction factor.

### Neutrophil-endothelial interaction by intravital retinal imaging

Female C57BL/6 mice, 10–12 weeks old weighing 20–30 g were purchased from Jackson Laboratories (Bar Harbor, ME), housed to a maximum of 5 per cage, and given ad libitum access to food and water. The experimental protocols were approved by the Institutional Animal Care and Use Committee of Massachusetts General Hospital (N° 2009N000085). Mice were injected retro-orbitally (r.o.) with 5 μg of rat anti-Ly6G-Alexa Fluor 647 (BioLegend, San Diego, CA) diluted in sterile PBS as a single 50 μL bolus 3 h before inducing endotoxemia. Endotoxemia was induced by injecting 3 mg/kg LPS i.p. (O55:B5, Sigma, USA). The treatment group was injected carvacrol 80 mg/kg i.p. in a single 75 μl bolus 1 h after inducing endotoxemia. Serial intravital imaging was conducted at times 3, 6, 12, 24, 48, and 72 h. Humane endpoint was accomplished by anesthetic overdose followed by cervical dislocation.

For intravital imaging mice were anesthetized using 3% isoflurane and placed on a thermoregulated stage adapted on a scanning laser ophthalmoscope (SLO) [39, 40]. A diode laser with 638 nm wavelength (Micro Laser Systems, Inc. Garden Grove, CA, USA) was used to detect reflected light and to excite AF647 fluorescence. A spinning polygon scanner (Lincoln Laser Corp., Phoenix, AZ) and a galvanometric mirror (GSI Lumonics, Billerica, MA) raster scan a field of view of 425 to 575 μm on the retina at video-rate (30 frames per second). Reflectance images were acquired by separating the backscattered from incident light by means of polarization, using a quarter-wave plate and a polarizing beam splitter cube. Light reflected from the retina was focused through a confocal pinhole (diameter 25 μm ≈ 1.25 airy disc sizes) and detected by a photomultiplier tube (PMT) (R3896, Hamamatsu, Japan). Fluorescence emanating from the retina was transmitted into a dedicated fluorescence detection arm through a dichroic beamsplitter (Di03-R405/488/532/638, Semrock, Rochester, NY), and detected through a 650 nm longpass filter (Semrock, Rochester, NY) and a confocal pinhole (diameter 50 μm ≈ 2.5 airy disc sizes) with a PMT (R3896, Hamamatsu, Japan). Neutrophil-endothelium interactions (NEI) were operationally defined as neutrophil-endothelium interactions over 1 s. Baseline intravital imaging (detailed below) was performed 2 h before inducing endotoxemia. At each timepoint, multiple 30 s videos were captured in a region close to the optical nerve head. NEI was quantified from SLO videos by visual inspection and the count of NEI was normalized per minute. Noise was removed from movies with 3D Median filter in ImageJ/Fiji. From the processed movies, five frames were averaged to generate a still frame for presentation.

### Determination of coagulation and blood flow changes induced by endotoxin in Zebrafish

Adult zebrafish were maintained at the fish facility of the Universidad Andres Bello, following standard protocols. Wild type AB (WT) and Tg(*fli1*:eGFP)^y1^ embryos were obtained by natural spawning and maintained at 28 °C in E3 medium (5 mM NaCl, 0.17 mM KCl, 0.33 mM CaCl_2_, 0.33 mM MgSO_4_, pH 7.0). Tg(*fli1*:eGFP)^y1^ (*fli1*:eGFP) is a transgenic line in which ECs and platelets are fluorescently labeled that allows to observe blood vessels and tracking platelet flow [41]. Furthermore, a *trpm7* crispant in a WT genetic background (*trpm7* crispant^*WT*^) embryos and a *trpm7* crispant in a *fli1*:eGFP genetic background (*trpm7* crispant^*fli1:eGFP*^) embryos, were generated as described below. Larval ages are expressed in days post fertilization (dpf). Handling procedures were approved by the Commission of Bioethics and Biosafety of Universidad Andres Bello (N° 004/2021).

WT, Tg(*fli1*:eGFP)^y1^, *trpm7* crispant^*WT*^ and *trpm7* crispant^*fli1:eGFP*^ 4 dpf larvae were anesthetized with 0.03% tricaine (Sigma, USA) and mounted in 1% low melting agarose (Sigma, USA) before microinjection. Using a microneedle attached to an air-driven Cell Tram (NARISHIGE, Japan), larvae were non-injected or individually microinjected in the heart with 20 nL of saline buffer (NaCl 0,9%) or endotoxin (LPS (O55:B5 Sigma, USA) 100 ng) or non-injected. To determine the existence of coagulation, 4 dpf WT or *trpm7* crispant^*WT*^ larvae were stained with o-dianisidine (0.6 mg/mL, 0.01 M sodium acetate pH 4.5, 0.65% H_2_O_2_, and 40% ethanol) for 30 min at RT, washed with distilled water and fixed in 4% PFA at 4 °C overnight. Larvae were imaged with a Leica MZ12.5 stereomicroscope and images were processed using ImageJ software and BioVoxxel plugin to quantify the total pixel intensity units (IU) in a defined region of interest (ROI). To determine changes in blood flow, time-lapse assay were performed on 5 dpf Tg(*fli1*:eGFP)^y1^ or *trpm7* crispant^*fli1:eGFP*^ larvae using a Leica TCS Sp8 confocal microscope acquiring images every 2 s for 60 s in the segment of the caudal vein above the midgut. Images were processed using ImageJ software.

### CRISPR-Cas9-mediated genome editing.

Guide RNAs (gRNAs) design: to generate F0 *trpm7* knockout zebrafish embryos (*trpm7* crispants) [42], four gRNAs targeting different exons of the *trpm7* gene were designed using CHOPCHOP [43–45] and CRISPRscan [46] webtools (Additional file 1: Table S1). Each gRNA was generated using two oligonucleotides; a tail primer (5’-AAAAGCACCGACTCGGTGCCACTTTTTCAAGTTGATAACGGACTAGCCTTATTTTAACTTGCTATTTCTAGCTCTAAAAC-3’) and a gRNA primer containing the T7 promoter sequence and part of the *trpm7* coding sequence (Additional file 1: Figure S1). Both primers were annealed by PCR to generate a DNA template using the following program: initial denaturation at 95 °C for 10 secs, followed by 40 cycles of 95 °C for 10 secs, 60 °C for 10 secs and 72 °C for 10 secs. Then, each DNA template was transcribed using the mMESSAGE mMACHINE T7 kit (Ambion, ThermoFisher) to generate the corresponding gRNA, which was assessed for size and quality on an electrophoresis gel.

Cas9/gRNA complex assembly and injection: A mix of 120 ng/μL of gRNAs (30 ng/μL each RNA), 300 ng/μL NLS-Cas9 protein (PNA Bio Inc, USA) and 1 μl enzyme buffer (PNA Bio Inc, USA) was incubated at 37 °C for 5 min prior to microinjection. Then, 1-cell stage WT or Tg(fli1:eGFP)^y1^ embryos were microinjected with 2 nL of the Cas9/gRNA mix (0.24 ng gRNAs and 0.6 ng NLS-Cas9 per embryo). As control, not injected embryos or injected with the same mix mentioned above but using the saCas9 null mutant NLS protein (ABM) which will bind to genomic DNA but will not introduce any genome modifications were used. *Trpm7* crispant^*WT*^ and *trpm7* crispant^*fli1:eGFP*^ larvae were identified phenotypically at 3 dpf by a strong reduction in size and number of cutaneous pigments, as previously reported for *trpm7* mutant fish [47].

### Detection of vWF, ICAM-1, P-Selection and NF-κB by fluorescent immunocytochemistry

Cultured ECs were washed twice with PBS and fixed with 3.7% paraformaldehyde (PFA) for 30 min at RT before being permeabilized with 0.1% Triton X-100 in PBS for 30 min at RT and then blocked for 2 h at RT with 3% BSA in PBS. Cells were washed again and incubated with the primary antibodies to detect endothelial vWF, ICAM-1, P-Selection and NF-κB (Abcam). Then, cells were washed twice and incubated with the secondary antibodies. Samples were mounted with ProLong Gold antifade mounting medium with DAPI (Invitrogen, USA). For the quantification of fluorescence, areas of interest were selected and subjected to analysis using the Image J software. Fluorescence quantification was normalized against control condition (vehicle-treated condition).

### Platelet-Neutrophil Aggregates (PNAs) formation assay

Endothelial cells were plated at a density of 2.5 × 10^4^ cells in a 96-well plate, and after 24 h were transfected with anti TRPM7 or control siRNA. To start the assay, LPS (O127:B8, Sigma-Aldrich) was added at a final concentration of 20 µg/mL for 24 h at 37 °C with fluorescent-labeled platelets (2.25 × 10^6^ platelets per well, contained in 100 µL of minimal experimentation medium) and 30 min before finishing the experiment, pre-isolated and -stained neutrophils (~ 2.5 × 10^4^ per well contained in 100 µL of minimal experimentation medium) were added. Finally, unattached cells (platelet and neutrophils) were removed by three careful washes with PBS. Attached platelets, neutrophils, and aggregates formation were determined using fluorescence microscopy. The analysis of aggregates was made by counting of close or merged spots in every field.

### Rats and experimental groups. blood samples and primary mesenteric endothelial cell isolation

Male Sprague–Dawley rats (180–210 g) were housed in cages with water and food ad libitum, a 12 h light/dark cycle and 25 ± 1 ºC temperature. All experimental protocols were approved by the Commission of Bioethics and Biosafety from Universidad Andres Bello (N°002/2020). Rats were separated into 4 groups: Group 1: AdV^*CTRL*^-injected + saline-treated group. Rats were subjected to *i.v.* injections of AdV encoding RFP used as a control sequence in 300 µL. After 30 min, rats were injected *i.p.* with 100 µL sterile saline solution (N = 16). Group 2: AdV^*CTRL*^-injected + endotoxin-treated group. Rats were subjected to *i.v.* injections of AdV^*CTRL*^ in 300 µL. After 30 min, rats were injected with endotoxin (LPS (0127:B8, Sigma, USA), 10 mg/kg) in 100 µL. (N = 16). Group 3: AdV^*shTRPM7*^-injected + saline-treated group. Rats were subjected to *i.v.* injections of AdV encoding an shRNA against TRPM7 expression in 300 µL. After 30 min, rats were injected *i.p.* with 100 µL sterile saline solution (N = 16). Group 4: AdV^*shTRPM7*^-injected + endotoxin-treated group. Rats were subjected to *i.v.* injections of AdV^*shTRPM7*^ in 300 µL. After 30 min, rats were injected with endotoxin (LPS, 10 mg/kg) in 100 µL. (N = 16). All AdV were injected at 3 × 10^10^ viral particles.

Rat blood samples were collected in a sodium citrate blood collection Vacutainer^®^ tube through cardiac puncture after anesthetizing. Obtained blood was immediately centrifuged at 4,000 rpm for 10 min at 4 °C to separate the plasma, which was immediately stored at −80ºC. Rat mesenteric endothelial cells (RMECs) were isolated from the mesenteric artery. The mesenteric artery was occluded on its distal end and cannulated from its proximal end with a polyethylene tubing connected to a 21-gauge syringe. The mesentery was surgically removed and washed with sterile PBS. For the enzymatic isolation of RMECs, each mesenteric artery was slowly perfused in a culture hood for 5 min with 5 mL M-199 medium supplemented with 40 μL Pen/Strep 10,000 U/mL/10,000 μg/mL), 20 μL Fungizone (250 μg/mL), and 12.5 mg collagenase type II. The cell suspension was centrifuged at 3,000 rpm for 7 min; the pellet was reconstituted in 3 mL M-199 medium supplemented with 8 mL/L of Pen/Strep (10,000 U/mL/10,000 μg/mL), 4 mL/L of Fungizone (250 μg/mL), 10% FBS, and 10% CCS. Thereafter, the cells were subjected immediately to experiments.

### Adenovirus production and infection

For complementary alignment, the primers (FWR: 5` TCGAGGCACCTTTATATCATTATTCAAGAGATAATGATATAAAGGTGCCTTTTT-3’.

REV: 5’-GAAAAAGGCACCTTTATATCATTATCTCTTGAATAATGATATAAAGGTGCC-3’) were diluted to 100 µM in a buffer solution containing (in mM) 50 NaCl, 10 Tris–HCl, 10 MgCl_2_ and 10 µg/mL BSA, pH 7.9 and incubated at 95 °C for 5 min followed by 3 h incubation at 25 °C. The shRNA was then subcloned into the pShuttleU6 vector (Addgene) using *XbaI* and *SalI* (New England Biolabs). The adenoviral vectors were generated using the AdEasy system. Briefly, homologous recombination was carried out by electroporation of BJ5183 cells (Agilent Technologies) with *PmeI* linearized DNA (pAD/RFP adenovirus) was used as control. Recombinant adenoviral plasmids were digested with *PacI* (New England Biolabs) and transfected into AdHEK293 cells with Lipofectamine 2000 (Life Technologies) according to the manufacturer’s guidelines. Following observation of cytopathic effects for 21 days, the cells were scraped and subjected to four freeze–thaw cycles in a dry-ice methanol bath. The resulting supernatant was used to infect a 10 cm dish of 70% confluent AdHEK293 cells. Following observation of CPEs after 5–7 days, viral particles were purified and expanded by infecting 4 plates of AdHEK293 cells. For specificity and efficiency see Additional file 1.

### Platelet aggregation assay, platelet count, d-dimer determination, and bleeding and clotting times assay

Platelet aggregation was measured using a four-channel platelet aggregometer (Chrono-Log Corp., USA) after the addition of 0.1 μg/mL collagen or 2 μM ADP. PRP was incubated for 5 min at 37 °C. After incubation, platelet aggregation was determined as the level of light transmission monitored for 5 min. Platelet aggregation was expressed as percentage of the platelet-poor plasma (PPP) transmission values. Bleeding time was measured by the tail transection model. After treatment, the tail tip was transected with a scalpel at a point that measured 1 mm in diameter. Bleeding was assessed every 30 s using a filter paper, and the time to clot formation defined the bleeding time. Clotting time was determined by collecting blood into three separated test tubes prewarmed at 37 °C to obtain an average result. Blood clotting was tested by tipping the tube back and forth every 30 s, and clotting time was determined when blood does not flow out from tubes when tilted horizontally. Platelet count was determined using a blood cell counter HEMAVET950 (Drew Scientific, USA). d-dimer level determination was performed in plasma by use of Rat d-Dimer ELISA Kit according to instructions by the manufacturer (Abbexa, UK).

### Determination of mRNA and protein TRPM7 expression

RT-qPCR experiments were performed to measure TRPM7 mRNA levels. Total RNA was extracted with Trizol according to the manufacturer's protocol (Invitrogen, Carlsbad, CA). DNAse I-treated RNA was used for reverse transcription using the Super Script II Kit (Invitrogen, Carlsbad, CA). Equal amounts of RNA were used as templates in each reaction. Quantitative-PCR was performed using the SYBR Green PCR Master Mix (AB Applied Biosystems, Foster City, CA). Assays were run using a RotorGene instrument (Corbet Research, Sydney, Australia). Data are presented as relative mRNA levels of the gene of interest normalized to relative levels of 28S mRNA and normalized against control condition.

Flow cytometry analysis was performed to determine expression changes in TRPM7 expression using monoclonal antibodies (Abcam), coupled to suitable secondary antibodies conjugated to fluorophores (ThermoFisher). The labeled cells were then analyzed immediately by flow cytometry (BD FACS Fortessa, BD Biosciences, San José, CA). Color compensation matrices were calculated for each staining combination within each experiment using single-stained antibody. In all analyses, doublets and clusters were eliminated. A minimum of 10,000 events were analyzed.

### Liver and kidney markers determination

Blood extraction was performed by cardiac puncture into lithium heparin-containing tubes. The blood was immediately centrifuged at 4,000 rpm for 10 min at 4 °C to isolate the plasma. To measure markers of liver and kidney failure, we quantified plasma levels of aspartate aminotransferase (AST), alanine aminotransferase (ALT), total bilirubin (TBIL) for liver function, and creatinine (CRE) and blood urea nitrogen (BUN) for kidney function. Markers were quantified using the Piccolo Xpress Chemistry Analyzer (General Chemistry 13, MetLyte Plus CRP and Basic Metabolic Panel Plus panels, Abaxis, USA) according to manufacturers' instructions. Plasma urea was determined by DiaSys Urea CT FS (DiaSys Diagnostic Systems, Holzheim, Germany), according to the manufacturers’ instructions. Plasma levels of kidney injury molecule-1 (KIM-1), neutrophil gelatinase-associated lipocalin (NGAL) and β2-microglobulin (β2M) markers were quantified by corresponding rat ELISA Kit (Abcam).

### Shock septic patients and volunteers

The study was conducted in 22 shock-septic patients (SSPs) and 25 healthy volunteers (HVs), admitted to the intensive care unit (ICU) at Hospital Clínico Metropolitano La Florida (HLF) located in Santiago, Chile. Additional file 1: Table S2 shows the clinical and demographic patient characteristics. SSPs features are shown in Additional file 1: Tables S3. This study was approved by the HLF Institutional Ethics and Bioethics Review Board (N°141,008). Additionally, the Commission of Bioethics and Biosafety of Universidad Andres Bello also approved all experimental protocols (N°002/2020). The investigation conforms with the principles outlined in the Declaration of Helsinki. All participants or their surrogates signed an informed consent form prior to entry into the study.

Inclusion criteria for SSPs include age > 18 y.o. without limitation for resuscitation and suffering from shock defined operationally as a requirement for a norepinephrine (NE) dose > 0.1 g⋅kg^−1^⋅min^−1^ to maintain the median arterial pressure (MAP) between 65 and 80 mmHg and a lactate concentration > 4 mM. Furthermore, patients had respiratory support with invasive mechanical ventilation and a C-reactive protein level ≥ 15 mg/dL. These criteria had to be met 48 h after patients were admitted to the ICU.

Exclusion criteria for SSPs comprised consumption of drugs that modify coagulation, fibrinolysis, and platelet aggregation in the last 14 days. Also excluded were patients with solid cancer with a more advanced stage than carcinoma in situ, organ transplantation, leukemia, lymphoma, pregnancy, liver cirrhosis, nephrotic syndrome, chronic dialysis, congestive heart failure and red blood cell transfusion with > 2 units within the last 48 h after ICU admission. SSPs with chemotherapy, hospitalization or surgery within the last 3 months prior to ICU admission were also excluded.

HVs were enrolled from the outpatients in the hospital area and research facilities at Universidad Andres Bello. The abovementioned exclusion criteria were also applied to the HVs. The operational definition of HVs was people without any known chronic disease and explicitly without arterial hypertension, chronic allergic condition, diabetes, body mass index > 30 kg/m^2^, smoking, pregnancy and coagulation dysfunction. In addition, those subjects with an episode of hospitalization or surgery in the last 3 months prior to enrollment in the study were excluded.

Demographic, clinical and laboratory data were carefully recorded and collected. The APACHE II score was evaluated after admission to the ICU and SOFA score was determined on the day of blood recollection. Management and treatment of patients was carried out by the attending physicians at ICU as per the standard of care and without any specific intervention for the purpose of this study. Twenty-eight-day mortality was also recorded.

### CECs separation from SSPs and protein expression determination by flow cytometry

Circulating endothelial cells (CEC), including circulating endothelial mature cells (CMECs) and circulating endothelial progenitor cells (CEPCs) were separated from blood samples obtained from SSPs 48 to 72 h after admission to the ICU, and from HVs. Blood samples were collected in a 3 ml vacutainer tube containing EDTA as anticoagulant. Collection of blood samples and isolation of cells and their analysis were carried out by double-blinded personnel, who were totally blinded to the patient group corresponding to the collected sample, as well as clinical characteristics or further outcome of the patients.

The CMECs and CEPCs were isolated by magnetic bead-based immunoseparation as described previously [48–50]. Briefly, after blood samples were obtained, total mononuclear blood cell fraction was isolated from blood by Ficoll-Histopaque (Sigma-Aldrich, USA) gradient separation. The mononuclear cell fraction was washed by centrifugation with phosphate-buffered saline solution. Then, the mononuclear blood cell fraction was subjected to immunomagnetic bead capture (IBC) using a bead-conjugated CD133 monoclonal antibody and magnetic cell separation system (Miltenyi Biotec). The captured cells corresponded to an enriched CEPC sample (positive selection, CD133^+^), while the cells in the eluted solution contained CMECs (negative selection, CD133^−^). To directly isolate CMECs, eluted fluid was subsequently subjected to a second step of IBC positive selection using a bead-conjugated CD146 monoclonal antibody (Miltenyi Biotec), obtaining an enriched CMECs sample (CD146^+^ and CD133^−^). CMEC and CEPC quantification was performed by flow cytometry. Compensation particles (BD CompBeads) and amine polymer microspheres (Becton Dickinson) were used for compensation [48–50]. Fluorescently conjugated antibodies against VE-Cadherin^+^ and CD31^+^ and against VEGFR-2^+^ and CD34^+^ were used for the detailed phenotype characterization of CMECs and CEPCs, respectively.

### Statistical analysis

Results are presented as mean ± SEM or mean ± 95% confidence interval (CI) for the relative risk. Differences were considered significant at *p* < 0.05. Statistical differences were assessed by student’s t-test (Mann–Whitney type) to compare two groups, one-way analysis of variance (one-way ANOVA or Kruskal–Wallis type) followed by Dunn’s post hoc test to compare more than the two groups and test one independent variable, or two-way analysis of variance (two-way ANOVA) followed by Tukey’s or Dunnett’s post hoc test to compare more than the two groups and test two independent variables. See the figure legends for the specific test used. The relationships between variables were assessed by means of correlation analysis using Spearman’s correlation coefficients and linear regression. Survival Kaplan–Meier curves were compared by Log-rank (Mantel-Cox) test and Gehan-Breslow-Wilcoxon test to determine survival rates. Contingency analyses with Fisher’s exact test were used to assess the relative risk of death. The ability of TRPM7 expression to predict death at 28-days was assessed using the area under the receiver operating characteristic curve (AUROC) [51] with a 95% confidence interval (95% CI). Statistical testing was two-sided and used the 5% significance level. The data were analyzed with GraphPad Prism version 9.4 (GraphPad Software). Samples used in the study were defined to identify the mean magnitude effect of > twofold increase in TRPM7 expression between HVs and SSPs with standard deviations of 10%. Accordingly, a sample size of 22 SSPs and 25 HVs would provide 90% statistical power to detect an increase of TRPM7 expression level of > twofold using a two-sided 0.05 significance level.

## Results

### Platelet and neutrophil adhesion to ECs is mediated through TRPM7 under endotoxic conditions.

**Fig. 1:**
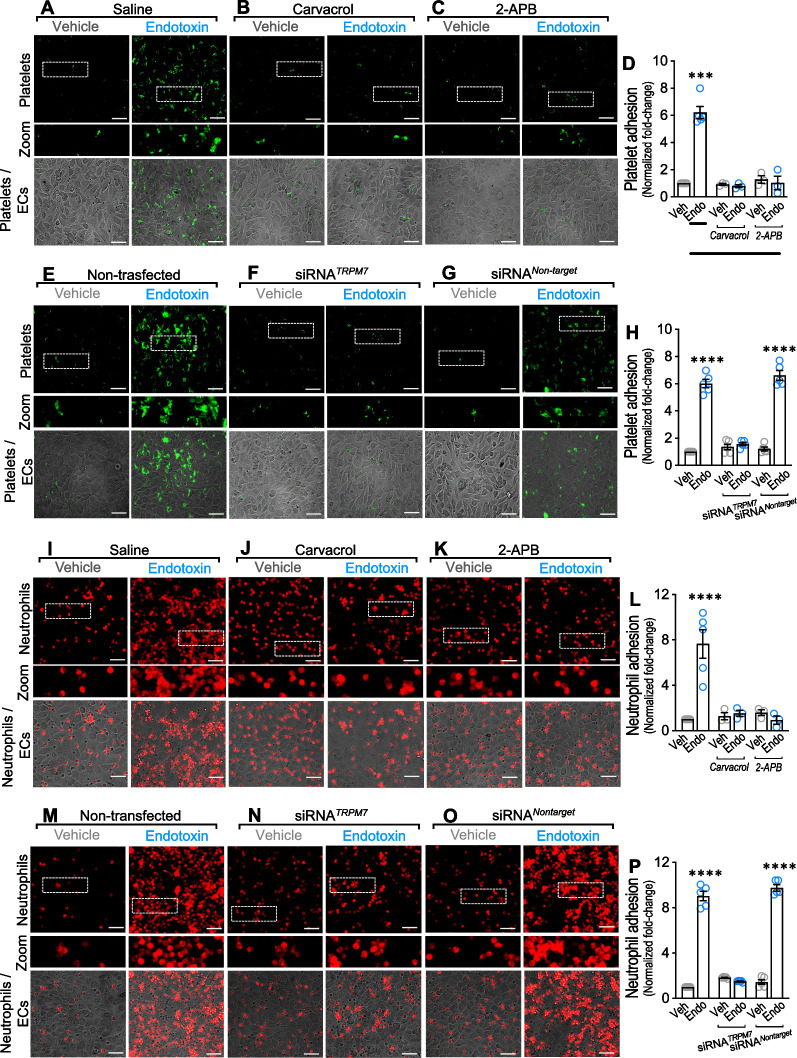
TRPM7 modulates endotoxin-induced platelet and neutrophil adhesion to endothelial cells. Representative images from vehicle- and endotoxin-treated ECs exposed to saline solution (**A**), carvacrol (**B**), 2-APB (**C**), or non-transfected (**E**), transfected with a siRNA against TRPM7 (siRNA^*TRPM7*^) (**F**) or transfected with a siRNA control (siRNA^*Nontarget*^) (**G**) and then cocultured with platelets for 24 h. Scale bar represents 100 μm. Platelets were stained by Vybrant Dio (green) and platelet adhesion was analyzed (**D** and **H**). (N = 5). Representative images from vehicle- and endotoxin-treated ECs exposed to saline solution (**I**), carvacrol (**J**), 2-APB (**K**), or non-transfected (**M**), transfected with a siRNA against TRPM7(siRNA^*TRPM7*^) (**N**) or transfected with a siRNA control (siRNA^*Nontarget*^) (**O**) and then cocultured with neutrophils for 30 min. Scale bar represents 100 μm. Neutrophils were stained by Vybrant Did (red). Neutrophil adhesion was analyzed (**L** and **P**). (N = 5). Results were normalized against vehicle-exposed cells (control condition). Statistical differences were assessed by a two-way analysis of variance (ANOVA) followed by Tukey post hoc test. ***p < 0.001 and ****p < 0.0001, compared with the vehicle-treated condition in the saline- or non-transfected-condition. Results showed as mean ± SEM

Considering that ECs exposed to endotoxin promote extensive platelet adhesion [52], we examined whether endotoxin-induced platelet adhesion to ECs requires endothelial TRPM7 participation. To that end, we performed experiments in endotoxin-treated human ECs cocultured with isolated human platelets, and platelet-to-EC adhesion was measured. ECs treated with endotoxin for 24 h showed an ~ sixfold increase in platelet adhesion (Fig. 1A right panel and D) compared with vehicle-treated ECs (Fig. 1A left panels). However, ECs preincubated for 1 h and maintained throughout the experiment with the TRPM7 inhibitor carvacrol completely abolished the endotoxin-induced platelet adhesion (Fig. 1B right panel and D). Of note, carvacrol also activates TRPV3 channels [53]. Preincubation for 1 h and maintained throughout the experiment with the non-selective calcium channel inhibitor 2-APB showed similar results (Fig. 1C right panel and D). Because of TRPM7 activity as a Ca^2+^-permeable channel, we examined changes in the intracellular calcium level using the Ca^2+^-sensitive fluorescent dye Fluo-4. The endotoxin-induced Ca^2+^-increase required TRPM7 activity, as carvacrol- and 2-APB-treatment inhibited the calcium increase (Additional file 1: Figure S2A).

Next, to unequivocally demonstrate that endothelial TRPM7 is required for platelet-to-EC adhesion, we applied a molecular biological experimental strategy to downregulate TRPM7 expression using a small interfering RNA (siRNA) against the human TRPM7 (siRNA^*TRPM7*^). The efficiency of siRNA^*TRPM7*^ in downregulating TRPM7 expression was > 90% compared to that of ECs transfected with a non-targeting siRNA (siRNA^*Nontarget*^) or nontransfected ECs (Additional file 1: Figure S2B). Thus, experiments were performed in endotoxin-treated ECs transfected with siRNA^*TRPM7*^ or siRNA^*Nontarget*^, and platelet adhesion was determined. The results showed that siRNA^*TRPM7*^-transfected ECs exposed to endotoxin (Fig. 1F right panel and H), completely prevented the endotoxin-induced platelet adhesion (Fig. 1E right panel and H) compared with vehicle-treated ECs (Fig. 1E left panel and H) or siRNA^*Nontarget*^-transfected endotoxin-treated ECs (Fig. 1G right panel and H). Additionally, similar to the above results, the endotoxin-induced Ca^2+^-increase was dependent on TRPM7 expression since transfection with siRNA-TRPM7 abolished the calcium increase (Additional file 1: Figure S2C). Considering that level of TRPM7 in plasma membrane could affects intracellular calcium level determination, we addressed changes in level of TRPM7 in the plasma membrane. ECs exposed to endotoxin showed increased levels of TRPM7 at the plasma membrane, which were not affected by either carvacrol or 2-APB treatment (Additional file 1: Figure S2D) but limited by siRNA^*TRPM7*^ (Additional file 1: Figure S2E).

Since neutrophil adhesion to ECs is a further key step in thrombus formation [54], we wondered whether endotoxin-induced neutrophil adhesion to ECs requires endothelial TRPM7 participation. Endotoxin-exposed ECs cocultured with neutrophils for 30 min showed an ~ eightfold increase in neutrophil adhesion (Fig. 1I right panel and L) compared with vehicle-treated ECs (Fig. 1I left panel and L). Interestingly, preincubation with carvacrol (Fig. 1J right panel and L) and 2-APB (Fig. 1K right panel and L) completely prevented the endotoxin-induced neutrophil adhesion, suggesting that the inhibition of calcium signaling reduces the adhesion of neutrophils to ECs.

Furthermore, ECs were transfected with siRNA^*TRPM7*^ and siRNA^*Nontarget*^, after which adhesion was determined. The results showed that siRNA^*TRPM7*^ transfection in endotoxin-treated ECs (Fig. 1N right panel and P), totally prevented the endotoxin-induced neutrophil adhesion (Fig. 1M right panel and P) compared with vehicle treatment (Fig. 1M left panel and P) or siRNA^*Nontarget*^-transfection (Fig. 1O right panel and P) in ECs.

**Fig. 2:**
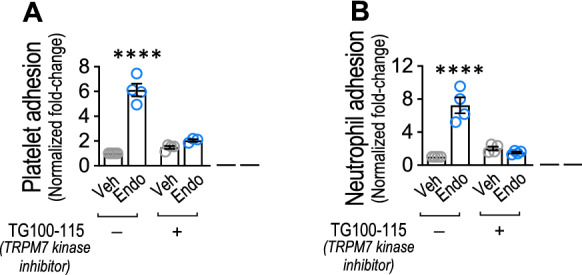
TRPM7 α-kinase domain participation in endotoxin-induced platelet and neutrophil adhesion to endothelial cells. Vehicle- and endotoxin-treated ECs exposed to saline solution or the TRPM7 α-kinase function inhibitor TG100-115, and then cocultured with platelets (**A**) or neutrophils (**B**) for 24 h. (N = 3–4). Results were normalized against vehicle-exposed cells (control condition). Statistical differences were assessed by a two-way analysis of variance (ANOVA) followed by Tukey post hoc test. ****p < 0.0001, compared with the saline-treated condition. Results showed as mean ± SEM

Next, we evaluated whether the TRPM7 α-kinase domain participates in the endotoxin-induced platelet and neutrophil adhesion to ECs. To that end, the TRPM7 α-kinase function inhibitor TG100-115 was used [55–57]. ECs pre-incubated for 1 h and maintained throughout the experiment with TG100-115 showed a significant decrease in the endotoxin-induced platelet (Fig. 2A) and neutrophil (Fig. 2B) adhesion when compared with endotoxin-induced platelet adhesion in the absence of TG100-115. These results suggest that TRPM7 α-kinase function is required for endotoxin-induced platelet and neutrophil adhesion to ECs.

### Neutrophil interactions with ECs are mediated through TRPM7 in endotoxemic mice.

**Fig. 3:**
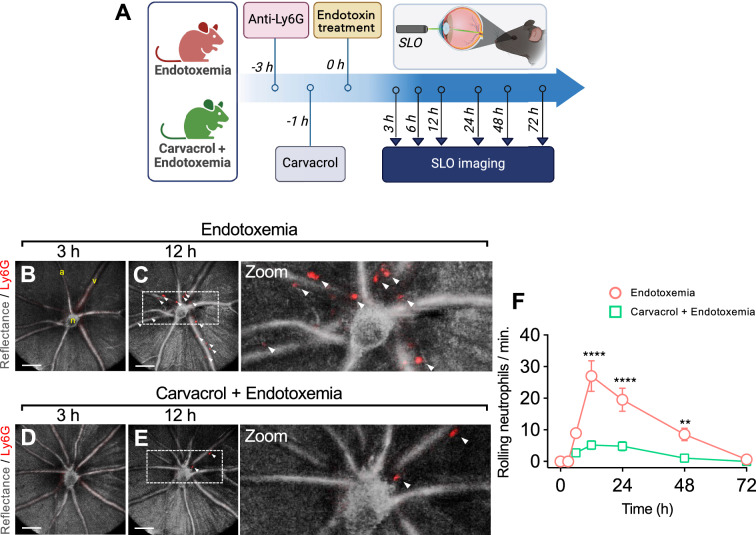
TRPM7 inhibition protects mice from increased neutrophil-endothelial interaction (NEI) during endotoxemia. **A** NEI in mice treated with LPS 3 mg/kg *i.p.* and or carvacrol 80 mg/kg i.p. (1 h post-endotoxemia) were inspected using a scanning laser ophthalmoscope (SLO) to obtain confocal images of the fundus from the left eye. **B–E** Representative SLO images obtained from a real-time movie depicting NEI count at 3 h (**B**) and 12 h (**C**) post-endotoxemia. Representative SLO images obtained from a real-time movie depicting NEI count at 3 h (**D**) and 12 h (**E**) post-endotoxemia in mice subjected to injection of carvacrol 1 h after endotoxemia. **F** Serial quantification of SLO captures from endotoxic mice in the absence or presence carvacrol at 0,3, 6, 12, 24, 48 and 72 h post-endotoxemia. Grey: reflectance with 638 nm laser. Red: rat anti-Ly6G. Arrowheads: neutrophils labelled with an anti-Ly6G IgG. v: Venules. a: Arterioles. n: Optic nerve head. Scale bar = 100 μm. (N = 5 per group). Statistical differences were assessed by two-way ANOVA followed by Dunnett's post hoc test. **p < 0.01, ****p < 0.0001 comparing endotoxemic (red) vs endotoxemic/carvacrol (green) conditions. Results showed as mean ± SEM

To evaluate the participation of TRPM7 in neutrophils rolling on blood vessels in an in situ model, intravital imaging experiments were performed in mice subjected to endotoxemia by *i.p.* administration of 3 mg/kg endotoxin in the presence or absence of 80 mg/kg carvacrol 1 h after endotoxemia induction (Fig. 3A). Because TRPM7 knockout in mice leads to early embryonic lethality [58], pharmacological modulation of TRM7 is the preferred approach. Confocal retinal reflectance imaging revealed the anatomical features that are characteristic of the retina: optic nerve head (n), arterioles (a), and venules (v) (Fig. 3B), which provide a morphological context to aid in following the trajectory and period of interaction of fluorophore-labeled neutrophils in situ.

Mice subjected to endotoxemia showed a transient increase in neutrophil-endothelial interaction (NEI), which increased starting at 6 h after endotoxemia induction (Fig. 3F), reached a peak at 12 h (Fig. 3C and F; Additional file 3: Movie S2) compared with 3 h (Fig. 3B and F; Additional file 2: Movie S1), and returned to baseline at 72 h (Fig. 3F). However, carvacrol administration 1 h after endotoxemia induction significantly attenuated the rise in NEI at the peak (Fig. 3E and F; Additional file 4: Movie S4) compared with 3 h (Fig. 3D and F; Additional file 4: Movie S3) and throughout the course of the experiment time-lapse (Fig. 3F). Neutrophil rolling is the final step in platelet-guided interaction with ECs. Thus, NEI is a decisive readout for studying endothelial activation during systemic inflammation, regardless of the degree of platelet contribution, yielding an approximate readout for detecting platelet interactions with ECs.

These results demonstrated that endotoxemic NEI can be suppressed in vivo by interfering with TRPM7 activation.

### Intravascular coagulation induced by endotoxin is mediated through TRPM7 in endotoxemic zebrafish.

**Fig. 4:**
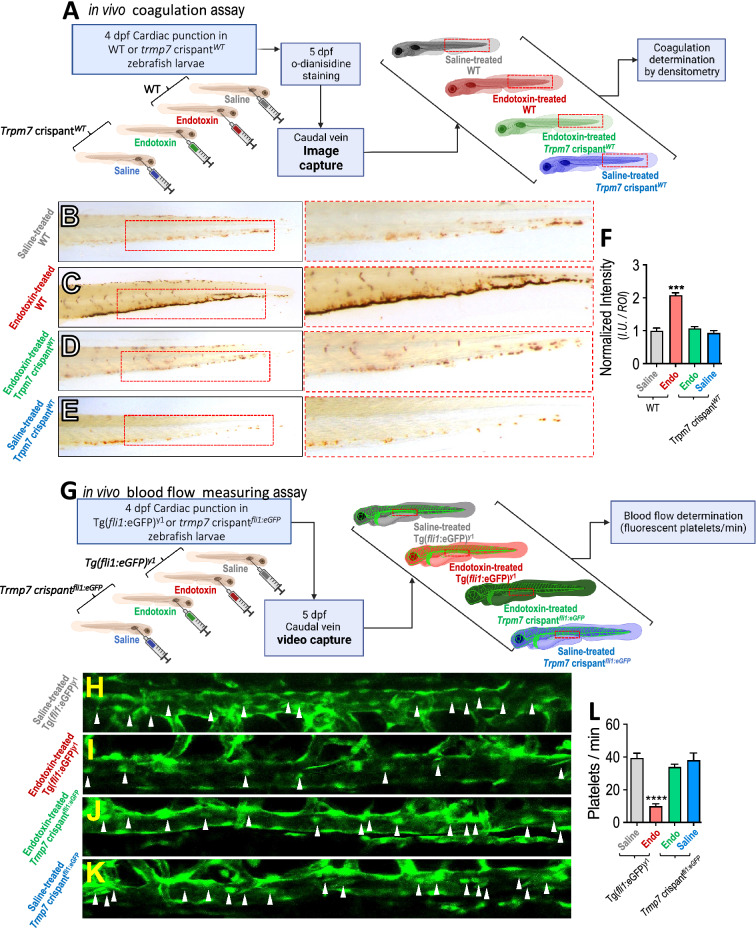
Administration of endotoxin induces coagulation in zebrafish vasculature mediated by TRPM7. (**A**) WT and *trpm7* crispant^*WT*^* zebrafish larvae* were subjected to o-dianisidine staining to evaluate in vivo coagulation. Larvae were injected with 20 nL sterile saline solution (NaCl 0,09%), or endotoxin (LPS (O55:B5 Sigma, USA) 100 ng). Thrombus formation was analyzed 24 h post injection in the caudal vein by o-dianisidine staining. (**B**–**E**) Representative images of saline-injected WT (**B**) endotoxin-injected WT (**C**), endotoxin-injected *trpm7* crispant^*WT*^ (**D**) and saline-treated *trpm7* crispant^*WT*^ conditions (**E**). Doted red box depicts o-dianisidine staining. **F** Quantification of o-dianisidine staining in caudal vein of *Zebrafish larvae* in saline-injected WT (grey bars), endotoxin-injected WT (red bars), endotoxin-injected *trpm7* crispant^*WT*^ (green bars) and saline-treated *trpm7* crispant^*WT*^ (blue bars) conditions. Results of the total pixel intensity (I.U.) in a defined region of interest (ROI), were normalized with the median value of saline condition. Tg(fli1:eGFP)^y1^ and *trpm7* crispant^*fli1:eGFP*^* zebrafish larvae*, having the vasculature and thrombocytes fluorescently green labeled, were subjected to time lapse analysis to evaluate blood flow in vivo coagulation. Blood flow time lapse analysis was determined as the number of platelets observed in 60 s in a section of the caudal vein (doted red box) were performed by time lapse analysis, in saline- and endotoxin-injected conditions (**G**). **H**–**K** Representative images of Tg(fli1:eGFP)^y1^ larvae saline-injected Tg(*fli1*:eGFP)^y1^ (**H**), endotoxin-injected Tg(*fli1*:eGFP)^y1^ (**I**), endotoxin-injected *trpm7* crispant^*fli1:eGFP*^ (**J**), and saline-treated *trpm7* crispant^*fli1:eGFP*^ conditions (**K**). **L** Quantification of blood flow time lapse analysis in a section of the caudal vein of Tg(*fli1*:eGFP)^y1^ *larvae* in saline-injected Tg(*fli1*:eGFP)^y1^ (grey bars), endotoxin-injected Tg(*fli1*:eGFP)^y1^ (red bars), endotoxin-injected *trpm7* crispant^*fli1:eGFP*^ (green bars) and saline-treated *trpm7* crispant^*fli1:eGFP*^ (blue bars) conditions. Statistical differences were assessed by a one-way analysis of variance (ANOVA) (Kruskal–Wallis) followed by Dunn's post hoc test. ***p < 0.001, ****p < 0.0001, compared with the saline-treated WT or Tg(*fli1*:eGFP)^y1^ conditions. Results showed as mean ± SEM

To determine the in vivo participation of TRPM7 in intravascular coagulation, a zebrafish model was used, which allows measurement of coagulation in the vasculature due to its transparency. To that end, *trpm7* crispant in a wild type AB (WT) genetic background (*trpm7* crispant^*WT*^) embryos were generated. Four days post fertilization (dpf) WT and *trpm7* crispant^*WT*^ zebrafish larvae were microinjected with saline solution or endotoxin. After microinjection, larvae were maintained for 24 h and then thrombus formation was analyzed using o-dianisidine staining in the caudal vein (Fig. 4A). The results showed that WT zebrafish larvae treated with endotoxin exhibited significant coagulation in the caudal vein compared to those treated with saline solution (Fig. 4B, C and F). However, endotoxin-injected *trpm7* crispant^*WT*^ zebrafish larvae showed decreased endotoxin-induced coagulation (Fig. 4D and F). Saline-injected *trpm7* crispant^*WT*^ does not cause any coagulation (Fig. 4E and F).

Next, we determined whether the existence of coagulation was connected to alterations in the blood flow. Thus, *trpm7* crispant in a Tg(*fli1*:eGFP)^y1^ (*fli1*:eGFP) genetic background (*trpm7* crispant^*fli1:eGFP*^) embryos were generated. Four dpf Tg(*fli1*:eGFP)^y1^ and *trpm7* crispant^*fli1:eGFP*^ zebrafish larvae, in which vasculature and platelets are fluorescently green labeled, were microinjected with saline solution or endotoxin. After 24 h, the number of platelets observed in 60 s interval was determined in vivo by time-lapse imaging in a section of the caudal vein (Fig. 4G). Tg(*fli1*:eGFP)^y1^ zebrafish larvae treated with endotoxin exhibited a significant reduction in the platelets flow (Fig. 4I and L, and Additional file 7: Movie 6) compared to those treated with saline-treated Tg(*fli1*:eGFP)^y1^ or saline-treated *trpm7* crispant^*fli1:eGFP*^ (Fig. 4H, K and L and Additional file 6: Movie 5 and Additional file 9: Movie 8). Interestingly, endotoxin-injected *trpm7* crispant^*fli1:eGFP*^ zebrafish larvae showed decreased endotoxin-induced reduction in blood flow (Fig. 4J and L; and Additional file 8: Movie 7).

Similar results were obtained in zebrafish larvae microinjected with saline solution or endotoxin in the presence or absence of the non-selective TRPM7 inhibitor FTY-720 (Additional file 1: Figure S3 and Additional file 10: Movie 9, Additional file 11: Movie 10, Additional file 12: Movie 11, Additional file 13: Movie 12).

### TRPM7 is required for endothelial vWF, ICAM-1 and P-selectin expression induced by endotoxin-mediated NF-κB activation

**Fig. 5:**
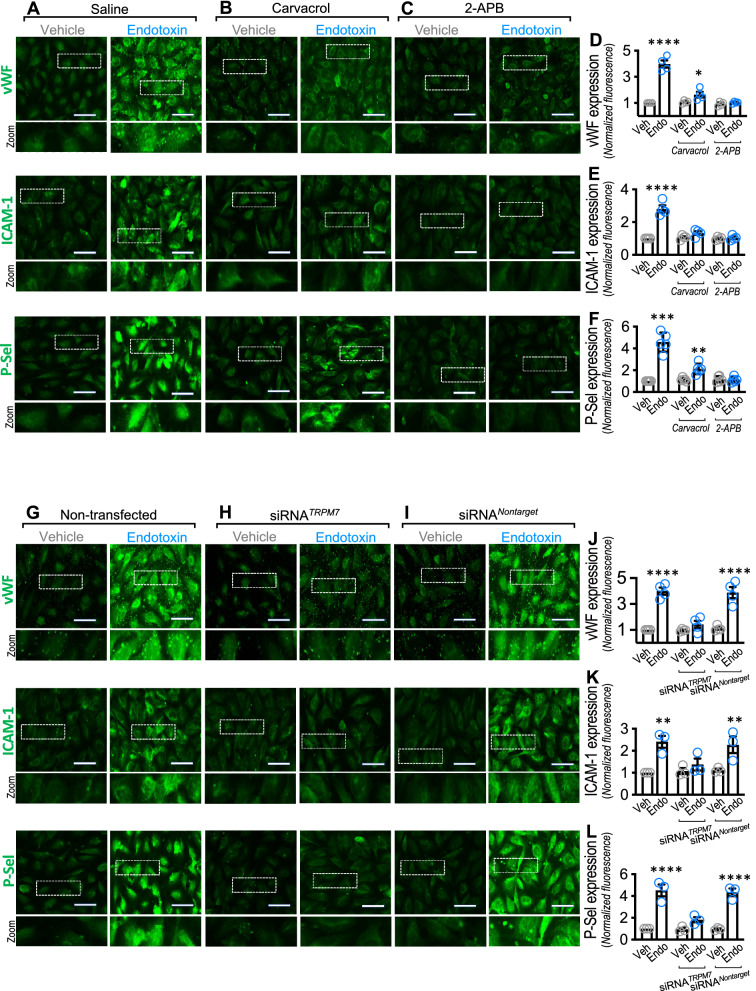
TRPM7 modulates endotoxin-induced vWF, ICAM-1 and P-Sel expression in endothelial cells. Representative images from vehicle- and endotoxin-treated ECs exposed to saline solution (**A**), carvacrol (**B**), 2-APB (**C**), or non-transfected (**G**), transfected with a siRNA against TRPM7 (siRNA^*TRPM7*^) (**H**) or transfected with a siRNA control (siRNA^*Nontarget*^) (**I**) for 24 h, and then vWF, ICAM-1 and P-Sel expression was detected by immunofluorescence. Bar scale represents 50 μm. Quantification of protein expression of vWF (**D** and **J**), ICAM-1 (**E** and **K**) and P-Sel (**F** and **L**) was performed by densitometric analysis (N = 5). Results were normalized against vehicle-exposed cells (control condition). Statistical differences were assessed by a two-way analysis of variance (ANOVA) followed by Tukey post hoc test. ***p < 0.001 and ****p < 0.0001, compared with the vehicle-treated condition in the saline- or non-transfected-condition. Results showed as mean ± SEM

It is well known that endothelial P-selectin, ICAM-1 and vWF are crucial proteins that promote platelet and neutrophil adhesion to ECs [17]. Thus, we studied whether TRPM7 is required for vWF, ICAM-1 and P-selectin expression in endotoxin-treated ECs. Endotoxin-treated ECs showed increased expression of vWF (Fig. 5A upper-right panel and D), ICAM-1 (Fig. 5A middle-right panel and E) and P-selectin (Fig. 5A lower-right panel and F), compared with the vehicle-treated condition. TRPM7 pharmacological inhibition with carvacrol showed significant prevention of endotoxin-induced vWF (Fig. 5B upper-left panels and D), ICAM-1 (Fig. 5B middle-left panels and E) and P-selectin (Fig. 5B lower-left panels and F) expression. Similarly, 2-APB incubation also showed significant prevention of endotoxin-induced vWF (Fig. 5C upper-left panels and D), ICAM-1 (Fig. 5C middle-left panels and E) and P-selectin (Fig. 5C lower-left panels and F) expression.

To directly assess whether TRPM7 is crucial for endotoxin-induced vWF, ICAM-1 and P-selectin expression, we performed experiments in siRNA^*TRPM7*^-transfected ECs. In siRNA^*TRPM7*^-transfected endotoxin-treated ECs, vWF (Fig. 5H upper-right panel and J), ICAM-1 (Fig. 5H middle-right panel and K) and P-selectin (Fig. 5H lower-right panel and L) expression was completely inhibited compared with that in endotoxin-treated non-transfected ECs (Fig. 5G) or siRNA^*Nontarget*^-transfected endotoxin-treated ECs (Fig. 5I).

Furthermore, endotoxin-induced mRNA expression of the tested adhesion proteins showed similar results. Endotoxin-induced mRNA expression of vWF, ICAM-1 and P-selectin (Additional file 1: Figure S4A–C), was inhibited by carvacrol and 2-APB treatment. Similarly, in siRNA^TRPM7^-transfected endotoxin-treated ECs, vWF, ICAM-1 and P-selectin mRNA expression was abolished (Additional file 1: Figure S4D–F).

**Fig. 6:**
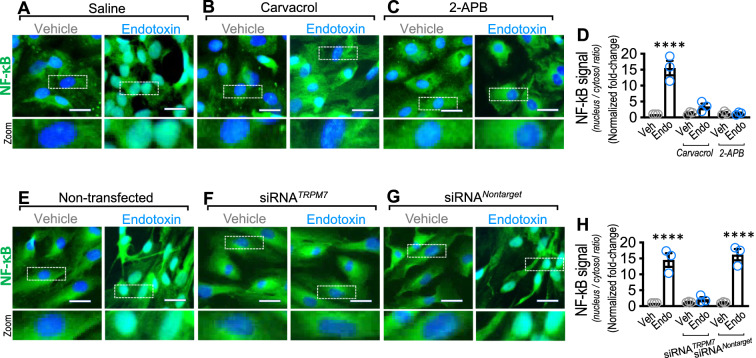
TRPM7 regulates endotoxin-induced translocation of NF- κB into the nucleus in endothelial cells. Representative images from vehicle- and endotoxin-treated ECs exposed to saline solution (**A**), carvacrol (**B**), 2-APB (**C**), or non-transfected (**E**), transfected with a siRNA against TRPM7 (siRNA^*TRPM7*^) (**F**) or transfected with a siRNA control (siRNA^*Nontarget*^) (**G**) for 24 h, and then NF-κB (green) was detected by immunofluorescence. Nuclei were stained using DAPI. Bar scale represents 50 μm. Quantification of NF-κB (**D** and **H**) was performed by densitometric analysis (N = 3). Data were expressed as the normalized ratio of NF-κB signal (pixels/μm^2^) in nucleus/cytosol. Results were normalized against vehicle-exposed cells (control condition). Statistical differences were assessed by a two-way analysis of variance (ANOVA) followed by Tukey post hoc test. ****p < 0.0001, compared with the vehicle-treated condition in the saline- or non-transfected-condition. Results showed as mean ± SEM

These results indicate that vWF, ICAM-1 and P-selectin expression induced by endotoxin requires transcriptional activation. The expression of vWF, ICAM-1 and P-selectin is induced by NF-κB activation in endotoxin-activated ECs [59–61]. Once endotoxin activates ECs, NF-κB translocates from the cytoplasm to the nucleus [62]. This translocation induces protein expression, including adhesion proteins such as vWF, ICAM-1 and P-selectin, supporting platelet and neutrophil adhesion to ECs [12]. Considering the abovementioned results showing that TRPM7 is required for endotoxin-induced P-selectin, ICAM-1 and vWF expression, we set out to investigate whether TRPM7 is also required for NF-κB translocation to the nucleus. The results showed that endotoxin-treated ECs depicted a strong and significant translocation of NF-κB to the nucleus compared with vehicle-treated ECs (Fig. 6A and E). However, endotoxin-treated ECs exposed to carvacrol (Fig. 6B) or 2-APB (Fig. 6C) or transfected with siRNA^TRPM7^ (Fig. 6F) showed inhibited endotoxin-induced NF-κB translocation. Consistently, densitometric analysis showed that the NF-κB nucleus/cytosol from endotoxin-treated ECs exposed to carvacrol and 2-APB (Fig. 6D) or transfected with siRNA^TRPM7^ (Fig. 6H) showed an inhibited endotoxin-induced NF-κB translocation.

### Participation vWF, ICAM-1 and P-selectin in endotoxin-induced platelet and neutrophil adhesion to ECs

**Fig. 7:**
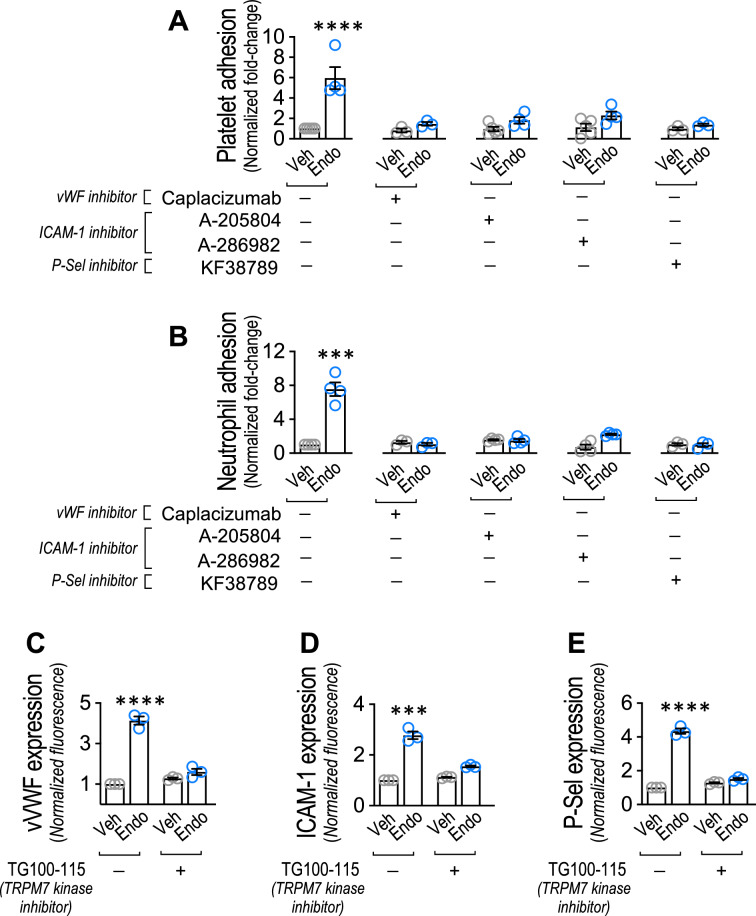
vWF, ICAM-1 and P-Sel participation in endotoxin-induced platelet and neutrophil adhesion to endothelial cells and TRPM7 α-kinase domain participation in endotoxin-induced vWF, ICAM-1 and P-Sel expression. **A**–**F** Vehicle- and endotoxin-treated ECs in the presence or absence of the vWF inhibitor Caplacizumab (**A**, **B**), the ICAM-1 inhibitor A-286982 and A-205804 (**C**, **D**), or the P-Sel inhibitor KF38789 (**E**, **F**) and then cocultured with platelets (**A**, **C** and **E**) or neutrophils (**B, D** and **F**) for 24 h. or 30 min. respectively, and platelets and neutrophils adhesion was analyzed (N = 3–4). (**G**–**I**) Protein expression quantification of vWF (**G**), ICAM-1 (**H**) and P-Sel (**I**) by densitometric analysis from immunofluorescence experiments in vehicle- and endotoxin-treated ECs in the presence or absence of the TRPM7 α-kinase function inhibitor TG100-115 (N = 3–4). Results were normalized against vehicle-treated cells in the absence of inhibitors (control condition). Statistical differences were assessed by a two-way analysis of variance (ANOVA) followed by Tukey post hoc test. ***p < 0.001 and ****p < 0.0001, compared with the vehicle-treated cells in the absence of inhibitors. Results showed as mean ± SEM

Next, we tested whether vWF, ICAM-1 and P-selectin participate in the endotoxin-induced platelet and neutrophil adhesion to EC. Pharmacological inhibition of the vWF, ICAM-1, and P-selectin proteins by means of the vWF inhibitor caplacizumab [63, 64], the ICAM-1 inhibitors A-286982 [65, 66] and A-205804 [67, 68], and the P-selectin inhibitor KF38789 [69, 70] was performed. The vWF inhibitor caplacizumab, the ICAM-1 inhibitors A-286982 and A-205804 and the P-selectin inhibitor KF38789 showed a significant inhibition in the endotoxin-induced platelets (Fig. 7A) and neutrophils (Fig. 7B) adhesion to ECs. These results suggest that vWF, ICAM-1, and P-selectin are required to complete the endotoxin-induced platelets and neutrophils adhesion process to EC-associated expression TRPM7.

Furthermore, we tested whether the TRPM7 α-kinase domain participates in the endotoxin-induced increased expression of vWF, ICAM-1, and P-selectin in ECs. ECs pre-incubated for 1 h and maintained throughout the experiment with the TRPM7 α-kinase function inhibitor TG100-115 led to a significant inhibition in the increased expression of vWF (Fig. 7C), ICAM-1 (Fig. 7D), and P-selectin (Fig. 7E) induced by endotoxin in ECs. These results suggest that TRPM7 α-kinase function is required for the endotoxin-induced increased expression of vWF, ICAM-1, and P-selectin in ECs.

### Platelet-neutrophil aggregate adhesion to ECs is mediated through TRPM7 under endotoxic conditions.

Platelet-neutrophil aggregates (PNAs) formation has been reported during sepsis and used as a marker of poor outcomes [71]. Considering the experiments described above showing separately that adhesion of both platelets and neutrophils was dependent on endothelial TRPM7, we were enticed to assess whether endothelial TRPM7 is involved in endotoxin-induced PNAs adhesion to ECs. For this purpose, isolated platelets (green) were cocultured for 24 h over a monolayer of endotoxin-treated human ECs, after which neutrophils (red) were added, and the tri-culture was incubated for 30 min. PNAs adhesion to ECs transfected with siRNA^*TRPM7*^ and siRNA^*Nontarget*^ was determined by close or merged green/red staining over the EC monolayer (see arrows).

**Fig. 8:**
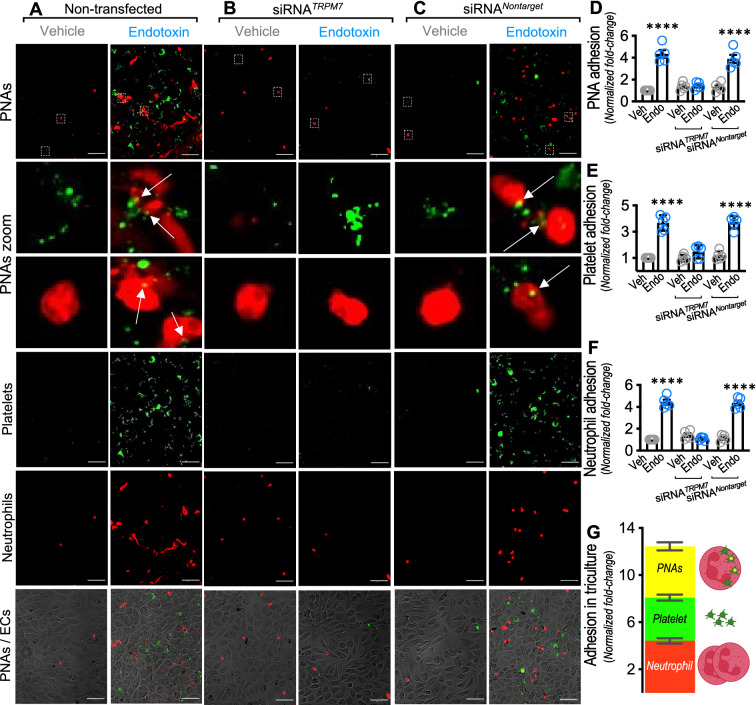
TRPM7 regulates endotoxin-induced PNAs formation an adhesion to endothelial cells. Representative images from vehicle- and endotoxin-treated ECs non-transfected (**A**), transfected with a siRNA against TRPM7 (siRNA^*TRPM7*^) (**B**) or transfected with a siRNA control (siRNA^*Nontarget*^) (**C**) cocultured with platelets for 24 h, and then, with neutrophils for 30 min. Platelets were stained by Vybrant Dio (green) and neutrophils by Vybrant Dio (red) and the level of adhesion of PNAs (**D**), platelets (**E**), neutrophils (**F**) to ECs was analyzed (N = 5). Bar scale represents 100 μm. Relative adhesion to ECs of PNAs, platelets and neutrophils in the triculture (**G**). PNAs aggregates on ECs were determined by close or merged green/red staining over the EC monolayer depicted by the arrows. Results were normalized against vehicle-exposed non-transfected cells (control condition). Statistical differences were assessed by a two-way analysis of variance (ANOVA) followed by Tukey post hoc test. *p < 0.05, **p < 0.01, ***p < 0.001 and ****p < 0.0001, compared with the vehicle-treated condition in the saline- or non-transfected-condition. Results showed as mean ± SEM

The results showed that endotoxin-treated non-transfected ECs exposed to platelets (green staining) and neutrophils (red staining) showed a ~ fourfold increase in PNAs (green/red staining) formation (Fig. 8A upper right-panel and D) compared with vehicle-treated non-transfected ECs (Fig. 8A upper left-panel and D). Interestingly, endotoxin-treated siRNA^*TRPM7*^ transfected ECs (Fig. 8B upper right-panel and D), completely prevented the endotoxin-induced PNAs aggregation compared with vehicle-treated non-transfected ECs (Fig. 8B upper left-panel and D). Results from endotoxin-treated siRNA^*Nontarget*^ transfected ECs (Fig. 8C upper left- and right-panels and D) showed similar results as those showed in non-transfected ECs. These results indicate that TRPM7 is required not only for either platelet or neutrophil adhesion but also for modulation of PNAs aggregates to endotoxin-treated ECs.

Interestingly, quantification of adhered platelets to ECs and adhered neutrophils to ECs which are not forming PNAs in the tri-culture, showed ~ 3.5-fold (Fig. 8E) and ~ fourfold (Fig. 8F) increased adhesion, respectively, which were ~ 2.5- and ~ fourfold lower than those shown in the coculture experiments (Fig. 1). Notably, such differences in platelet and neutrophil adhesion to ECs could be explained because platelets and neutrophils are captured in PNA-to-EC adhesion. (Fig. 8G).

### TRPM7 regulates the endotoxemia-induced procoagulant phenotype and death in endotoxemic rats, which is associated with liver and kidney failure

**Fig. 9:**
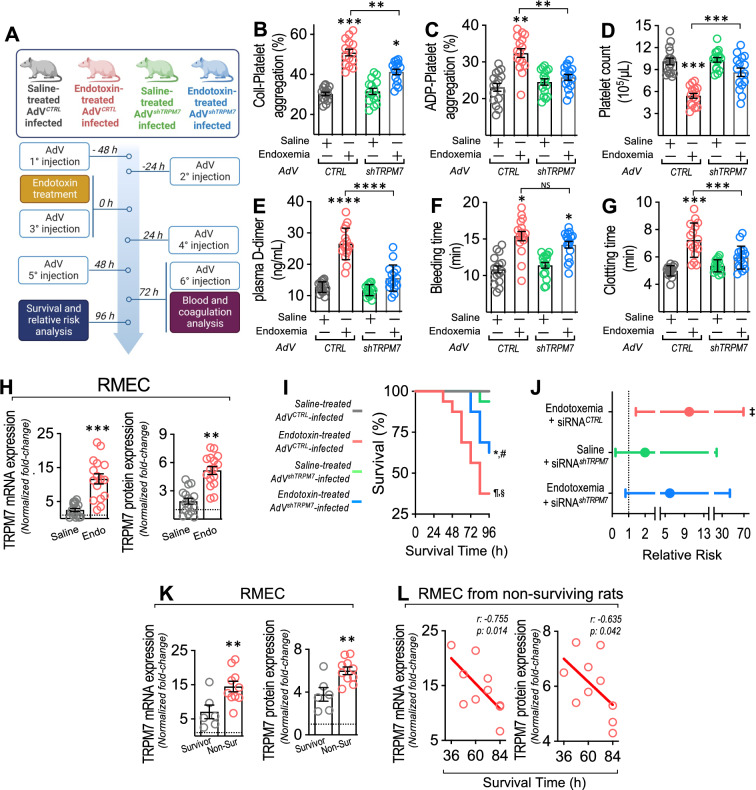
TRPM7 suppression protects from endotoxemia-induced procoagulant phenotype, increased death and risk of death during endotoxemia. **A** Experimental strategy for in vivo endotoxemia and AdV infection in rats. Rats were divided in 4 groups. Group 1: AdV^*CTRL*^-injected + saline-treated. Group 2: AdV^*CTRL*^-injected + endotoxin-treated. Group 3: AdV^*shTRPM7*^-injected + saline-treated. Group 4: AdV^*shTRPM7*^-injected + endotoxin-treated. Endotoxin was administrated at 10 mg/kg. AdVs were injected every 24 h from 48 before to 72 h after induction (6 injections). After treatment, blood samples were collected to determine collagen-induced platelet aggregation (**B**), ADP-induced platelet aggregation (**C**), platelet count (**D**), plasma D-dimer levels (**E**), bleeding time (**F**), and clotting time (**G**). AdV^*CTRL*^-injected + saline-treated (grey circles), AdV^*CTRL*^-injected + endotoxin-treated (red circles), AdV^*shTRPM7*^-injected + saline-treated (green circles), AdV^*shTRPM7*^-injected + endotoxin-treated (blue circles) rats. Statistical differences were assessed by one-way analysis of variance (ANOVA) followed by Dunn's post hoc test. *: *p* < 0.05, **: *p* < 0.01, ***: *p* < 0.001, ****: *p* < 0.0001, compared with the saline-treated condition. **H** TRPM7 mRNA and protein expression in RMEC from AdV^*CTRL*^-injected + saline-treated (Saline, grey circles) and AdV^*CTRL*^-injected + endotoxin-treated rats (Endo, red circles). Statistical differences were assessed by student’s t-test (Mann–Whitney) (N = 16). **p < 0.01, ***p < 0.001, compared with the survivor patients’ group. Results showed as mean ± SEM. **I** Survival (Kaplan–Meier) curves comparing AdV^*CTRL*^-injected + saline-treated (grey line) (N = 16), AdV^*CTRL*^-injected + endotoxin-treated (red line) (N = 16), AdV^*shTRPM7*^-injected + saline-treated (green line) (N = 16), AdV^*shTRPM7*^-injected + endotoxin-treated (blue line) (N = 16) rats. * and #, p = 0.002 (log-rank (Mantel–Cox) test) when comparing AdV^*CTRL*^-injected + endotoxin-treated versus AdV^*shTRPM7*^-injected + saline-treated condition (*) and AdV^*shTRPM7*^-injected + endotoxin-treated (#) rats. ¶ and §, p = 0.0008 (Gehan-Breslow-Wilcoxon test) when comparing AdV^*CTRL*^-injected + endotoxin-treated versus AdV^*shTRPM7*^-injected + saline-treated condition (¶) and AdV^*shTRPM7*^-injected + endotoxin-treated (§) rats. **J** Contingency analyses performed to determine relative risk between AdV^*CTRL*^-injected + endotoxin-treated (N = 16), AdV^*shTRPM7*^-injected + saline-treated (N = 16) and AdV^*shTRPM7*^-injected + endotoxin-treated (N = 16) rats showed in I. ‡, p < 0.05, compared with the AdV^*shTRPM7*^-injected + saline-treated condition. **K** TRPM7 mRNA and protein expression in RMEC from surviving (grey circles) and non-surviving (red circles) AdV^*CTRL*^-injected + endotoxin-treated rats. Statistical differences were assessed by student’s t-test (Mann–Whitney) (N = 16) **p < 0.01, compared with the survivor patients’ group. Results showed as mean ± SEM. **L** Correlation analyses between TRPM7 mRNA and protein expression with survival time in RMEC from non-surviving rats. The relationships between variables were assessed by means of correlation analysis using Spearman’s correlation coefficients and linear regression

To determine whether TRPM7 regulates the procoagulant phenotype induced by endotoxemia, we tested in rats if adenoviral-mediated suppression of TRPM7 expression reduces crucial coagulation parameters after endotoxemia generation by *i.p.* administration of endotoxin, after which several coagulation parameters were measured (Fig. 9A). Endotoxin administration was effective in inducing a septic phenotype in accordance with the accepted criteria for sepsis diagnosis in humans [1, 2]. Endotoxemic rats exhibited hypotension and tachycardia compared to saline-treated rats, indicating the generation of hemodynamic alterations consistent with sepsis (Additional file 1: Table S4). Additionally, plasma cytokines were increased, indicating the generation of sepsis-induced systemic inflammation (Additional file 1: Table S5).

Thus, loss-of-function experiments were performed to analyze the role played by TRPM7 in regulating the procoagulant phenotype during endotoxemia (Fig. 9A). To that end, TRPM7 expression was downregulated by adenoviral infection with an adenovirus (AdV) encoding a specific shRNA for TRPM7 (AdV^*shTRPM7*^) similar to a previous reported [72].

TRPM7 downregulation efficiency was evaluated in AdV^*shTRPM7*^-infected ECs, which showed > 95% downregulation (Additional file 1: Figure S5A-B), without a change in the expression of the TRPM7 homologue TRPM6 [73] and in the inflammation modulated channel TRPM2 [74], compared with AdV^*CTRL*^-infected rats used as a control at the dose used in this study (Additional file 1: Figure S5C). Furthermore, TRPM7 protein and mRNA expression were downregulated in fresh mesenteric endothelial cells (RMECs) extracted from AdV^*shTRPM7*^-infected rats compared with those from AdV^*CTRL*^-infected rats (Additional file 1: Figure S6). Considering that exogenous RNA may activate the innate response that upregulates interferon (IFN) genes through dsRNA-dependent protein kinase PKR [75, 76], analysis of plasma IFN-β levels was performed. Plasma IFN-β levels from AdV^*shTRPM7*^- and AdV^*CTRL*^-infected rats showed no increase in IFN-β (Additional file 1: Figure S7A). Consistent with these results, IFN-induced PKR expression levels in RMECs did not change in AdV^*shTRPM7*^- or AdV^*CTRL*^-infected rats (Additional file 1: Figure S7B). Finally, the survival of AdV^*shTRPM7*^- and AdV^*CTRL*^-infected rats was not significantly different from that of saline-treated rats in the timeframe used in this study (Additional file 1: Figure S7C), suggesting that siRNA administration did not affect rat survival.

Then, the participation of TRPM7 in the procoagulant phenotype during endotoxemia was investigated. The results showed that the increased levels of platelet aggregation induced by collagen and ADP observed in endotoxin-treated AdV^*CTRL*^-infected rats were significantly prevented in endotoxin-treated AdV^*shTRPM7*^-infected rats (Fig. 9B and C, respectively). Furthermore, the decreased platelet count measured in endotoxin-treated AdV^*CTRL*^-infected rats, which is consistent with aggregation-induced platelet consumption, was inhibited in endotoxin-treated AdV^*shTRPM7*^-infected rats (Fig. 9D, respectively). d-dimer is a marker of coagulation, as it increases as a consequence of fibrinolysis [77]. Endotoxin-treated AdV^*CTRL*^-infected rats showed increased plasma levels of d-dimer, whereas endotoxin-treated AdV^*shTRPM7*^-infected rats showed D-dimer plasma levels at control levels (Fig. 9E). Moreover, we evaluated the bleeding and clotting times since these are modified in the procoagulant phenotype in endotoxemic rats. Endotoxin-treated AdV^*CTRL*^-infected rats showed prolonged bleeding and clotting times, whereas endotoxin-treated AdV^*shTRPM7*^-infected rats showed normal clotting times while bleeding did not changed (Fig. 9F and G, respectively), a result that was consistent with platelet consumption. These results indicate that a procoagulant phenotype in endotoxemic rats requires TRPM7 expression.

Considering the abovementioned results, we wondered whether TRPM7 expression is increased in endotoxemia. Notably, TRPM7 mRNA and protein expression was enhanced in RMECs extracted from endotoxemic rats (Fig. 9H), suggesting increased expression in the endothelial vasculature. Considering that endotoxemic challenge induces mortality and that endotoxemia-induced endothelial TRPM7 expression increases, we were prompted to determine whether TRPM7 expression suppression protects against endotoxemia.

The survival curve within a 96 h time frame from endotoxin-treated AdV^*shTRPM7*^-infected rats showed significant survival protection compared with curves from endotoxin-treated AdV^*CTRL*^-infected rats, as indicated by the log-rank (Mantel‒Cox) test. To give more weight to deaths at early times, the Gehan-Breslow-Wilcoxon test was performed, which also showed that endotoxin-treated AdV^*shTRPM7*^-infected rats appeared to be protected from endotoxemia-induced death (Fig. 9I). Contingency analysis to determine the relative risk of death showed that endotoxin-treated AdV^*shTRPM7*^-infected rats showed no risk of death compared to the significant risk in endotoxin-treated AdV^*CTRL*^-infected rats (Fig. 9J). Interestingly, TRPM7 expression was significantly increased in RMECs extracted from non-surviving rats compared with RMECs from surviving rats (Fig. 9K). Of note, survival time in non-surviving rats strongly correlated with mRNA and protein TRPM7 expression (Fig. 9L).

**Fig. 10:**
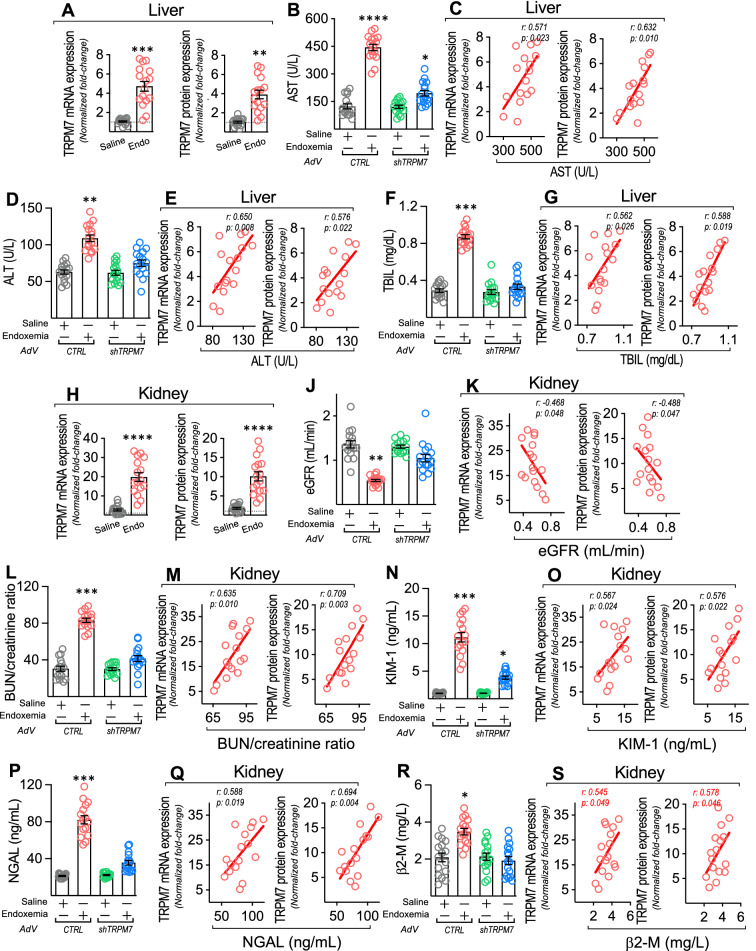
TRPM7 suppression protects from endotoxin-induced liver and kidney dysfunction, which correlates with TRPM7 expression. TRPM7 mRNA and protein expression in liver tissue from AdV^*CTRL*^-injected + saline-treated (Saline, grey circles) and AdV^*CTRL*^-injected + endotoxin-treated rats (Endo, red circles) (**A**). Statistical differences were assessed by student’s t-test (Mann–Whitney) (N = 16) **p < 0.01, ***p < 0.001, compared with the saline-treated condition. Results showed as mean ± SEM. Plasma liver dysfunction markers AST (**B**), ALT (**D**) and TBIL (**F**)**.** Statistical differences were assessed by one-way analysis of variance (ANOVA) followed by Dunn's post hoc test. (N = 16) **: *p* < 0.01, ***: *p* < 0.001, ****: *p* < 0.0001, compared with the saline-treated condition. Results showed as mean ± SEM. Correlation analyses between TRPM7 mRNA and protein expression with AST (**C**), ALT (**E**) and TBIL (**G**). The relationships between variables were assessed by means of correlation analysis using Spearman’s correlation coefficients and linear regression. TRPM7 mRNA and protein expression in liver tissue from AdV^*CTRL*^-injected + saline-treated (Saline, grey circles) and AdV^*CTRL*^-injected + endotoxin-treated rats (Endo, red circles) (**H**). Statistical differences were assessed by student’s t-test (Mann–Whitney) (N = 16) **p < 0.01, ***p < 0.001, compared with the saline-treated condition. Results showed as mean ± SEM. Plasma liver dysfunction markers eGFR (**J**), BUN/creatinine ratio (**L**) KIM-1 (**N**)**,** NGAL (**P**) and β2M (**R**)**.** Statistical differences were assessed by one-way analysis of variance (ANOVA) followed by Dunn's post hoc test. (N = 16) *: *p* < 0.05, **: *p* < 0.01, ***: *p* < 0.001, compared with the saline-treated condition. Results showed as mean ± SEM. Correlation analyses between TRPM7 mRNA and protein expression with eGFR (**K**), BUN/creatinine ratio (**M**) KIM-1 (**O**)**,** NGAL (**Q**) and β2M (**S**)**.** The relationships between variables were assessed by means of correlation analysis using Spearman’s correlation coefficients and linear regression

The procoagulant phenotype is strongly associated with organ dysfunction. During sepsis, DIC promotes organ dysfunction and is associated with increased death [78–81]. Considering this, we evaluated the participation of TRPM7 in liver and kidney dysfunction. Liver tissue from endotoxic rats showed increased TRPM7 mRNA and protein expression compared to saline-treated rats (Fig. 10A). Additionally, the results from the blood analysis showed that endotoxin-treated AdV^*shTRPM7*^-infected rats were protected from the endotoxic-induced increases in plasma levels of aspartate aminotransferase (AST) (Fig. 10B), alanine aminotransferase (ALT) (Fig. 10D), and total bilirubin (TBIL) (Fig. 10F) level, observed in endotoxin-treated AdV^*CTRL*^-infected rats, indicating that TRPM7 is required for liver failure during endotoxemia. Interestingly, the increased plasma levels of AST, ALT and TBIL in endotoxin-treated AdV^*CTRL*^-infected rats correlated with TRPM7 mRNA and protein expression (Fig. 10C, E and G respectively), supporting the notion that TRPM7 is associated with endotoxemia-induced liver failure.

Furthermore, the expression of TRPM7 mRNA and protein was increased in kidney from endotoxic rats compared to saline-treated rats (Fig. 10H). The glomerular filtration rate (GFR) is a well-accepted test for kidney malfunction [82]. Therefore, a validated plasma creatinine- and urea-based equation to determine the estimated GFR (eGFR) in male Sprague–Dawley rats was used [83]. First, we observed increased plasma creatinine and urea levels in endotoxin-treated AdV^*CTRL*^-infected rats, which was prevented in endotoxin-treated AdV^*shTRPM7*^-infected rats (Additional file 1: Figure S8). Endotoxin-treated AdV^*shTRPM7*^-infected rats showed protection against decreased GFR compared to that observed in endotoxin-treated AdV^*CTRL*^-infected rats (Fig. 10J). Correlation analysis showed that decreased GFR levels correlated with TRPM7 mRNA and protein expression in kidney tissue extracted from endotoxin-treated AdV^*CTRL*^-infected rats (Fig. 10K). Furthermore, the blood urea nitrogen/creatinine (BUN/creatinine) ratio was calculated as a kidney disease risk indicator. Endotoxin-treated AdV^*CTRL*^-infected rats showed an increased BUN/creatinine ratio, whereas endotoxin-treated AdV^*shTRPM7*^-infected rats exhibited protection against a BUN/creatinine ratio increase (Fig. 10L). Additionally, an increased BUN/creatinine ratio correlated with TRPM7 mRNA and protein expression in kidney tissue extracted from endotoxin-treated AdV^*CTRL*^-infected rats (Fig. 10M).

Next, we were prompted to delve deeper into kidney damage determination. Thus, the acute kidney injury (AKI) markers, kidney injury molecule-1 (KIM-1), neutrophil gelatinase-associated lipocalin (NGAL) and β2-microglobulin (β2M), were measured in plasma. Endotoxin-treated AdV^*CTRL*^-infected rats showed increased plasma KIM-1 (Fig. 10N), NGAL (Fig. 10P), and β2-M (Fig. 10R), indicating kidney dysfunction, while endotoxin-treated AdV^*shTRPM7*^-infected rats showed inhibition of increases in these markers. Additionally, correlation analysis showed that plasma KIM-1, NGAL and β2M levels correlated with TRPM7 mRNA and protein expression in kidney tissue extracted from endotoxin-treated AdV^*CTRL*^-infected rats (Fig. 10O, Q and S respectively).

These results indicate that TRPM7 suppression protects against endotoxic kidney failure.

### TRPM7 expression increases in circulating endothelial cells from SSPs, which is associated with DIC development

To study the participation of TRPM7 in septic shock patients and its association with DIC, we performed experiments in blood samples from 25 healthy volunteers (HVs) and 22 septic shock patients (SSPs). Additional file 1: Table S2 shows the clinical and demographic patient characteristics. SSPs features are shown in Additional file 1: Table S3.

**Fig. 11:**
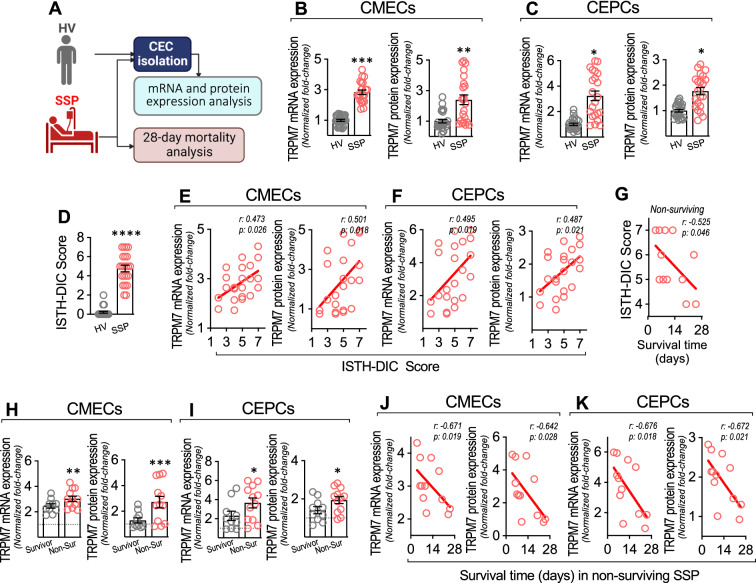
TRPM7 expression increases in CMECs and CEPCs from SSPs, which correlates with DIC and survival time. **A** Experimental strategy for SSPs (N = 22) and HVs (N = 25). TRPM7 mRNA and protein expression in CMECs (**B**) and CEPCs (**C**) extracted from HVs (grey circles) and SSPs (red circles). Statistical differences were assessed by student’s t-test (Mann–Whitney) (N = 16) *p < 0.05, **p < 0.01, ***p < 0.001, compared with the saline-treated condition. Results showed as mean ± SEM. Determination of ISTH-DIC Score in HVs (grey circles) and SSPs (red circles) (**D**). Statistical differences were assessed by student’s t-test (Mann–Whitney) (N = 16). ****p < 0.0001, compared with the HV group. Results showed as mean ± SEM. Correlation analyses between TRPM7 mRNA and protein expression with ISTH-DIC Score in CMECs (**E**) and CEPCs (**F**) extracted from SSPs. Correlation analyses between ISTH-DIC Score with survival time of non-surviving SSPs (**G**). The relationships between variables were assessed by means of correlation analysis using Spearman’s. TRPM7 mRNA and protein expression in CMECs (**H**) and CEPCs (**I**) extracted from surviving (grey circles) and non-surviving SSPs (red circles). Statistical differences were assessed by student’s t-test (Mann–Whitney) (N = 16) *p < 0.05, **p < 0.01, ***p < 0.001, compared with the survivor patients’ group. Results showed as mean ± SEM. Correlation analyses between TRPM7 mRNA and protein expression with survival time in CMECs (**J**) and CEPCs (**K**) of non-surviving SSPs. The relationships between variables were assessed by means of correlation analysis using Spearman’s

Considering that the vascular endothelium regulates thrombus formation, we performed experiments in circulating endothelial cells (CECs) from the SSPs. It has been reported that ECs from septic patients show increased blood CECs, which are composed of circulating mature endothelial cells (CMECs) and circulating endothelial progenitor cells (CEPCs) [48–50]. Magnetic bead-based immunoseparation was performed to successfully separate CMECs (CD146^+^, CD133^−^) and CEPCs (CD133^+^) using the appropriate markers (Fig. 11A and Additional file 1: Figure S9).

CMECs (Fig. 11B) and CEPCs (Fig. 11C) from the SSPs showed increased TRPM7 mRNA and protein expression compared to those of the HVs. To determine DIC incidence in patients, DIC score calculations are frequently used. Therefore, the DIC score was determined to evaluate its association with TRPM7 expression. To that end, the International Society on Thrombosis and Haemostasis (ISTH)-DIC score algorithm was used [79, 84] to determine DIC occurrence. The SSPs showed a significant DIC score compared with the HVs, with 13 patients with a DIC ≥ 5, which is considered as overt DIC (Fig. 11D). Correlation analysis showed that an increased DIC score correlated with enhanced TRPM7 mRNA and protein expression in CMECs (Fig. 11E) and CEPCs (Fig. 11F) obtained from SSPs. Interestingly, an increased DIC score correlated with a decreased survival time of non-surviving SSPs (Fig. 11G). Remarkably, non-surviving SSPs showed increased TRPM7 mRNA and protein expression in CMECs (Fig. 11H) and CEPCs (Fig. 11I), compared with surviving SSPs. Consistent with both, the correlations of DIC score with TRPM7 expression and of DIC score with survival time, enhanced TRPM7 mRNA and protein expression in CMECs (Fig. 11J) and CEPCs (Fig. 11K) correlated with decreased survival time of non-surviving SSPs. These results suggest that TRPM7 expression is associated with DIC establishment in SSPs.

### TRPM7 expression in circulating endothelial cells from SSPs is associated with death and DIC score and is a biomarker for predicting mortality in SSPs

**Fig. 12:**
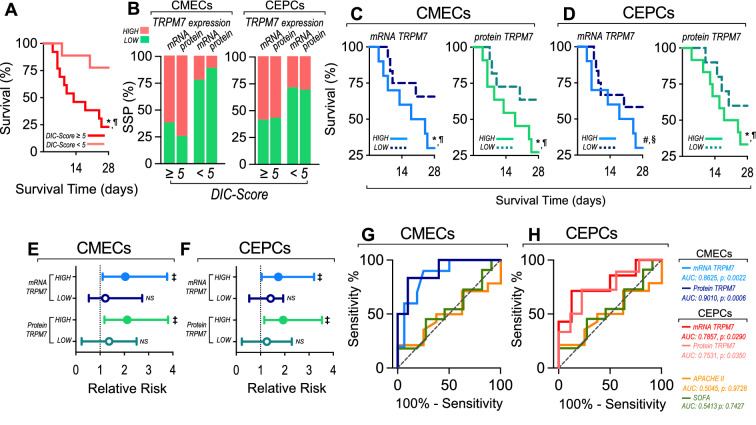
High TRPM7 expression increases in CMECs and CEPCs from SSPs, which correlates with DIC and survival time. **A** Survival (Kaplan–Meier) curves comparing SSPs exhibited a DIC-Score ≥ 5 (red line) with SSPs exhibited a DIC-Score < 5 (soft-red line). *, p = 0.0001 (log-rank (Mantel–Cox) test). ¶, p = 0.0002 (Gehan-Breslow-Wilcoxon test). **B** Percentage of SSPs exhibiting a DIC-Score ≥ 5 or DIC-Score < 5 showing high (red) and low (green) TRPM7 mRNA and protein expression in their CMECs and CEPCs. Survival (Kaplan–Meier) curves comparing high (solid soft-blue and soft-green) and low (dotted blue and green) TRPM7 mRNA and protein expression in CMECs (**C**) and CEPCs (**D**) with SSPs survival time. *, p = 0.0001 and #, p = 0.003 (log-rank (Mantel–Cox) test). ¶, p = 0.0002 and §, p = 0.007 (Gehan-Breslow-Wilcoxon test). Contingency analyses performed to determine relative risk between high (soft-blue and soft-green) and low (blue and green) TRPM7 mRNA and protein expression in CMECs (**E**) and CEPCs (**F**) respect to showed in C and D, respectively. ‡, p < 0.05, compared with the total (high and low together) mRNA and protein expression level of TRPM7. AUROC curve analysis in CMECs (**G**) and CEPCs (**H**) extracted from SSPs were performed for TRPM7 mRNA and protein expression. AUC and p-value score are: CMECs: AUC = 0.8625, *p* = 0.0022 (95% CI 0.7199–0.999) for TRPM7 mRNA expression and AUC = 0.9010, *p* = 0.0006 (95% CI 0.7702–1.000) for TRPM7 protein expression. CEPCs: AUC = 0.7857, *p* = 0.0290 (95% CI 0.5925–0.9789) for TRPM7 mRNA expression and AUC = 0.7531, *p* = 0.0350 (95% CI: 0.5638–0.9424) for TRPM7 protein expression. APACHE II: AUC = 0.5045, *p* = 0.9728 (95% CI: 0.2596–0.7493). SOFA: AUC = 0.5413, *p* = 0.7427 (95% CI 0.2934–0.7892). Diagonal discontinuous line in plots is the non-discrimination line. The best sensitivity (S) and specificity (E) to predict mortality outcome in SSPs and the corresponding Youden INDEX (YI) are: TRPM7 mRNA expression in CMECs: S: 90,00%, E:75,00%, YI:0.68, cut-off ≥ 2.698; TRPM7 protein expression in CMECs: S:83.33%, E:90.00%, YI:0.67, cut-off ≥ 2.299; TRPM7 mRNA expression in CEPCs: S:71,43%, E:87,50%, YI:0.61, cut-off ≥ 2.947; TRPM7 protein expression in CEPCs: S:72.22%, E:77.78%, YI:0.59, cut-off ≥ 1.825; APACHE-II: S:50.00%, E:62.50% and YI:0.28, cut-off ≥ 22.7 and SOFA: S:54.55%, E: 54.55% and YI:0.32, cut-off ≥ 8.3

The survival curve within a 28-day time frame from SSPs with a DIC score ≥ 5 showed a significantly decreased survival compared with the curve from SSPs with a DIC score < 5, as indicated by Log-rank (Mantel-Cox) test and Gehan-Breslow-Wilcoxon test (Fig. 12A).

Next, TRPM7 mRNA and protein expression was separated into high- and low-TRPM7 expression groups in both CMECs and CEPCs, using the corresponding median expression value as the threshold for separating the groups. Interestingly, the high-TRPM7 expression groups of the CMECs (Fig. 12B, left panel) and CEPCs (Fig. 12B, right panel) showed a high proportion of SSPs with a DIC score ≥ 5.

Survival curve analyses at 28 days were performed in SSPs separated into high- and low-TRPM7 expression groups to determine differences in mortality. The results showed that non-surviving SSPs with a high TRPM7 expression in CMECs (Fig. 12C) and in CEPCs (Fig. 12D), showed significant differences compared to the low-expression group of patients, as indicated by Log-rank (Mantel-Cox) test and Gehan-Breslow-Wilcoxon test.

Contingency analysis to determine the relative risk of death showed that SSPs with high- TRPM7 expression in CMECs (Fig. 12E) and in CEPCs (Fig. 12F), showed an increased risk of death compared to the low-TRPM7 expression SSPs group.

To estimate the capacity to predict mortality by measuring TRPM7 expression in CECs from the SSPs, we performed an area under the receiver operating characteristic curve (AUROC) analysis. TRPM7 expression from CMECs (Fig. 12G) and CEPCs (Fig. 12H) from the SSPs showed a high predictive capacity showing AUROC values higher than the diagonal nondiscrimination line, which supports their statistical significance. Both, the TRPM7 mRNA and protein expression in CMECs (Fig. 12G) showed better predictive values than those in CEPCs (Fig. 12H). Notably, in CMECs and CEPCs, mortality prediction based on TRPM7 expression was better when compared with the APACHE-II and SOFA scores, respectively, which are scores of severity and prognosis, respectively, widely used for critically ill patient evaluations (Fig. 12G–H).

These results indicate that the TRPM7 level in CMECs is associated with SSPs mortality and as increased risk of death and appears to be a useful and more accurate biomarker for predicting mortality in SSPs.

## Discussion

The endothelium is recognized as a key regulator of hemostasis since it releases several anticoagulant and fibrinolytic molecules that preserve vascular integrity [85]. In sepsis, ECs actively participate in the initiation of hemostatic disorders such as DIC, a life-threatening condition characterized by widespread thrombus formation promoting deficiencies in tissue perfusion with a subsequent impact on organ failure and mortality [86]. Given the close association between exacerbated platelet- or neutrophil-endothelium adhesion and the occurrence of thrombosis [87], the comprehensive study of these interactions is pivotal for improving septic patient outcome [88, 89]. ECs exhibit high platelet adhesion under conditions of endotoxin stimulation, which is consistent with previous studies describing that prestimulated platelets attach to intact HUVEC monolayers [90], or that platelets are recruited to murine intestinal venular ECs after an endotoxin challenge [91] and adhere to murine lung endothelium after endotoxin inhalation [92].

Here, we showed that the procoagulant phenotype during endotoxemia is mediated by TRPM7 ion channel and α-kinase activity in ECs, which promotes liver and kidney failure and increases both death and the relative risk of death. Interestingly, TRPM7 expression levels in CMECs and CEPCs from SSPs patients exhibited a significant capacity to predict mortality.

Calcium signaling plays a major role in sepsis progression and has been considered as a therapeutic target since clinical evidence supports the use of blockers of several types of calcium channel, such as slow N-type, L-type and T-type voltage-dependent calcium channels, dihydropyridine calcium channel, and stromal interaction molecule 1 (STIM1)-mediated store-operated Ca^2+^ entry (SOCE), among others, for improving the outcome of septic patients [93–97]. In fact, clinical and experimental data show that calcium signaling participates in several endothelial-driven events, such as endothelial fibrosis [98, 99], hyperpermeability [26] or senescence [100]. Importantly, our results suggested the participation of the intracellular calcium level to the adhesion of platelets to endothelial monolayers and, specifically, the contribution of TRPM7 to this process. This finding is remarkable, since in previous studies we demonstrated that TRPM7 mediates the increase in intracellular calcium concentration after an endotoxin challenge in ECs [98], and supports previous contributions describing the central role of TRPM7 in the regulation of endothelial function [101].

Likewise, neutrophils have emerged as strong contributors to sepsis-induced DIC, given their increased stiffness, reduced transmigration and increased high affinity attachment to the endothelium in inflammatory environments [102]. Our results showed that under conditions of endotoxemia, neutrophils firmly adhere to the endothelium, which is consistent with previous studies describing increased neutrophil adhesion in sinusoids after endotoxin stimulation [103]. The role of calcium signaling in neutrophil function has been previously shown, and the role of TRPM7 in neutrophil function under conditions of endotoxin stimulation was recently described in which it was shown to be a master regulator of migration [55].

TRPM7 contains a cytosolic α-kinase domain that participates in several function arterial thrombosis [30–32, 35]. Interestingly, TRPM7 α-kinase function has been reported as essential for neutrophil recruitment and function [55], suggesting that the TRPM7 ion channel activity and the α-kinase function are both important for controlling cellular functions.

Inspection of complex cell dynamics using intravital microscopy is a powerful tool [104]. While other approaches involve invasive surgical procedures to expose vascular beds [105, 106], noninvasive inspection of the retinal vasculature enables serial captures on the same individual over time without disturbing the tissular landscape. To the best of our knowledge, this is the first time that TRPM7 has been reported to modulate neutrophil-to-endothelial interactions in situ.

PNA formation is well documented in septic environments, and the role of platelets as a guide for neutrophil adhesion has been reported [107]. This is interesting, considering that individual platelet and neutrophil adhesion to ECs was higher compared with adhesion of platelets and neutrophils (in PNAs) to the endothelium. This could provide some clues about interactions between platelets and neutrophils before adhering to the endothelium, promoting a possible consumption of these cells. Moreover, we found altered morphology in some neutrophils in the tri-cultures exposed to endotoxin, which could suggest the formation of neutrophil extracellular traps. However, this process and the possible involvement of TRPM7 need further exploration.

Considering the observed expression of vWF, ICAM-1 and P-selectin after endotoxin stimulation, it is feasible to suggest that the adhesion of platelets and neutrophils is mediated by those adhesion proteins. It has been reported that neutrophils and platelets can simultaneously bind to the endothelium through P-selectin [108]. However, the theory of participation of ICAM-1 and vWF in simultaneous adhesion has been discarded [108]. Conversely, in separate studies, ICAM-1 has been shown to be involved in neutrophil adhesion and transmigration through the activated endothelium [109] but has also been associated with platelet adhesion to the endothelium. In the same study, the role of vWF in the adhesion of platelets was established [90], whereas the involvement of vWF in neutrophil recruitment in septic mice was also demonstrated [110]. The abovementioned findings illustrate the controversial participation of endothelial adhesion proteins in the adhesion of platelets and neutrophils. Therefore, further explorations in this field are needed.

Interestingly, we also observed that TRPM7 inhibition abolishes the increased expression of ICAM-1, P-selectin and vWF induced by endotoxin, and thus, the transcriptional machinery seems to play a relevant role, considering that TRPM7 inhibition also reduced the translocation of NF-κB induced by endotoxin. Although there are no previous findings linking calcium signaling induced by TRPM7 with NF-κB activity, it has been demonstrated that intracellular calcium is essential for NF-κB activity in murine neonatal neurons [111]. Furthermore, in T cells, it was demonstrated that calcium mediates the phosphorylation of p65, increasing the expression of inflammatory cytokines [112].

Interestingly, it has been reported that increased circulating levels of endoglin is observed in septic patients [113, 114]. Endoglin is an endothelial membrane glycoprotein that regulates transforming growth factor-β-mediated vascular function that also exhibits a circulating form and is markedly upregulated upon inflammation [115, 116]. Notably, endoglin is involved in hemostasia regulation via platelet adhesion to ECs [117] and in vascular pathologies, such as the hereditary hemorrhagic telangiectasia [118, 119]. Further studies are required to investigate the potential association between circulating endoglin or endoglin receptor and TRPM7 in the endotoxin-induced platelet and neutrophil adhesion to ECs.

Sepsis-induced DIC correlates with renal and liver failure [120–122], suggesting that this condition is associated with a prothrombotic environment. Notably, the increase in DIC parameters in endotoxic rats was blunted by TRPM7 expression suppression, indicating that TRPM7 mediates the prothrombotic phenotype during endotoxemia. Coagulopathy is associated with acute kidney injury (AKI), increasing the risk of death and liver failure in patients with sepsis [122, 123], while anticoagulant therapy in septic patients with AKI was shown to decrease the 28-day mortality rate [124]. Our results showed that TRPM7 expression suppression protects against liver and kidney dysfunction in endotoxemic rats, indicating that TRPM7 mediates prothrombotic actions in those organs. Consistent with these results, the risk of death and mortality decrease upon TRPM7 expression suppression. These findings are in agreement with those showing that TRPM7 expression is linked to liver fibrosis and kidney injury [26, 28, 125]. Notably, the endotoxemia-induced increases in the AKI markers KIM-1, NGAL and β2M were inhibited by TRPM7 suppression. KIM-1 and NGAL are markers of tubular damage [126–129]**.** Furthermore, β2M is linked to kidney diseases because almost all plasma β2M is excreted by the kidneys. However, since blood β2M is influenced by nonrenal factors [130], it requires supplementation with urine values or further determinations to associate it with kidney damage. Thus, TRPM7 overactivation could modify the hemostatic balance in the kidney, promoting dysfunction. Decreased GFR indicates kidney malfunction, and consistent with this, intraglomerular coagulation explains this GFR decrease and, consequently, the increased risk of death and mortality. Furthermore, the BUN/creatinine ratio is considered a consistent assay for detecting kidney dysfunction [131]. A high BUN/creatinine ratio is associated with acute kidney injury. Here, the BUN/creatinine ratio was increased in endotoxemic rats, indicating an increased risk of kidney disease. Dehydration, gut bleeding, thyroid malfunction and muscle disease can modify the BUN/creatinine ratio [132, 133]. Our results did not show skeletal muscle damage, indicating that creatinine is not influenced by this parameter. Additionally, autopsy did not show internal bleeding. We ruled out thyroid malfunction, considering the time course of the experiments, and dehydration, as water consumption was checked constantly.

SSPs showed enhanced TRPM7 expression in CMECs and CEPCs. These findings agree with those shown in blood samples from septic patients [36], although these previous results showed a TRPM7 increase that was several-fold lower than that shown in our experiments. However, in this study, TRPM7 expression was determined in serum samples without assessing the associated cellular fraction, making it difficult to elucidate the cellular source of TRPM7. Additionally, this study failed to explore the functional actions of TRPM7. Interestingly, the calculated DIC score in the SSPs, which determines the establishment of DIC in the SSPs, showed a strong association with TRPM7 expression in CMECs and CEPCs, suggesting that the DIC score in the SSPs could be mediated by enhanced TRPM7 activity. This idea is reinforced considering that enhanced TRPM7 expression in CMECs and CEPCs is associated with decreased survival time in non-surviving SSPs and that TRPM7 expression increases more in non-surviving SSPs than in surviving ones.

Interestingly, SSPs with a high TRPM7 expression in CMECs and CEPCs showed a marked increase in mortality compared to SSPs with a low TRPM7 expression, which exhibited an increased risk of death. Of note, AUROC analysis showed that TRPM7 expression in CMECs and CEPCs exhibited a significant capability for predicting mortality. In line with these findings, the notion that TRPM7 expression could serve as a predictor of mortality in septic patients was previously reported [36]. Determination of TRPM7 expression in CMECs, rather than in CEPCs, exhibits more accurate results for predicting mortality. Remarkably, this capacity for predicting mortality was better than the broadly used scores of severity and organ failure, such as APACHE-II and SOFA scores, respectively. Thus, TRPM7 expression in CMECs emerges as a new diagnostic tool for predicting mortality in SSPs, which could be more accurate than APACHE-II and SOFA scores.

## Conclusions

To our knowledge, this is the first comprehensive study that shows molecular evidence from in vitro to in vivo experiments to understand the crucial role of TRPM7 in DIC progression and its impact in DIC-mediated organ dysfunction and death during sepsis. TRPM7 mediates DIC progression, and its activity is required in DIC-induced organ dysfunction during sepsis. TRPM7 ion channel activity and the α-kinase function are required for endotoxin-induced increased platelet and neutrophil adhesion to ECs and for the endotoxin-induced increased expression of vWF, ICAM-1, and P-selectin. Importantly, endotoxin-induced expression of vWF, ICAM-1 and P-selectin were required for endotoxin-induced platelet and neutrophil adhesion to ECs. TRPM7 associates with increased mortality and enhanced risk of death during sepsis. Notably, TRPM7 expression appears to be a new prognostic tool for predicting mortality. Therefore, TRPM7 emerges as a novel target for treating DIC in SSPs since its suppression promotes increased survival and decreased risk of death during sepsis.

## Supplementary Information

**Additional file 1. **Supplemental information.

**Additional file 2. **Representative time lapse of retinal circulation from endotoxic mouse (LPS 3 mg/kg i.p.) at 3 h showing neutrophil-endothelial (NEI).

**Additional file 3. **Representative time lapse of retinal circulation from endotoxic mouse (LPS 3 mg/kg i.p. ) at 12 h showing neutrophil–endothelial interaction (NEI).

**Additional file 4. **Representative time lapse of retinal circulation from endotoxic mouse (LPS 3 mg/kg i.p.) at 3 h showing neutrophil-endothelial interaction (NEI) in the presence of Carvacrol (80 mg/kg i.p., 1 h post-endotoxemia).

**Additional file 5. **Representative time lapse of retinal circulation from endotoxic mouse (LPS 3 mg/kg i.p.) at 12 h showing neutrophil-endothelial interaction (NEI) in the presence of Carvacrol (80 mg/kg i.p., 1 h post-endotoxemia).

**Additional file 6. **Representative time lapse of Tg(fli1:eGFP)y1 4 dpf larvae microinjected in the heart with 20 nL of saline buffer (NaCl 0,9%).

**Additional file 7. **Representative time lapse of trpm7 crispant fli1:eGFP 4 dpf larvae microinjected in the heart with 20 nL of saline buffer (NaCl 0,9%).

**Additional file 8. **Representative time lapse of Tg(fli1:eGFP)y1 4 dpf larvae microinjected in the heart with 20 nL of endotoxin (LPS 100 ng).

**Additional file 9. **Representative time lapse of trpm7 crispantfli1:eGFP 4 dpf larvae microinjected in the heart with 20 nL of endotoxin (LPS 100 ng).

**Additional file 10. **Representative time lapse of Tg(fli1:eGFP)y1 4 dpf larvae microinjected in the heart with 20 nL of saline buffer (NaCl 0,9%).

**Additional file 11. **Representative time lapse of Tg(fli1:eGFP)y1 4 dpf larvae microinjected in the heart with 20 nL of endotoxin (LPS 100 ng).

**Additional file 12. **Representative time lapse of Tg(fli1:eGFP)y1 4 dpf larvae microinjected in the heart with 20 nL of endotoxin (LPS 100 ng) and treated by immersion with the TRPM7 inhibitor FTY-720 (0.5 ng/μL).

**Additional file 13. **Representative time lapse of Tg(fli1:eGFP)y1 4 dpf larvae microinjected in the heart with 20 nL of saline buffer (NaCl 0,9%) and treated by immersion with the TRPM7 inhibitor FTY-720 (0.5 ng/μL).

## Data Availability

The data used to support the findings of this study are available from corresponding author upon request.

## Abbreviations

ACD: Acid-citrate-dextrose
AKI: Acute kidney injury
APACHE II: Acute Physiology and Chronic Health Evaluation II
AdV: Adenovirus
AdV^*shTRPM7*^: Adenovirus encoding a specific shRNA for TRPM7
AdV^*CTRL*^: Adenovirus encoding a control shRNA for TRPM7
ANOVA: Analysis of variance
2-APB: 2-Aminoethoxydiphenyl borate
AUROC: Area under the receiver operating characteristic
β2M: β2-Microglobulin
BUN: Blood urea nitrogen
BUN/creatinine: Blood urea nitrogen/creatinine
CECs: Circulating ECs
CEPCs: Circulating endothelial progenitor cells
CMECs: Circulating mature endothelial cells
CRE: Creatinine
CI: Confidence interval
dpf: Days post fertilization
DIC: Disseminated intravascular coagulation
DMEM-Low: Dulbecco’s Modified Eagle Medium Low
ECs: Endothelial cells
FBS: Fetal bovine serum
GFR: Glomerular filtration rate
gRNAs: Guide RNAs
HVs: Healthy volunteers
HUVEC: Human umbilical vein endothelial cell
ICAM-1: Intercellular adhesion molecule-1
ISTH: International Society on Thrombosis and Haemostasis
ICU: Intensive care unit
IU: Intensity units
IFN: Interferon
KIM-1: Kidney injury molecule-1
LPS: Lipopolysaccharide
MAP: Median arterial pressure
NEI: Neutrophil-endothelium interactions
NGAL: Neutrophil gelatinase-associated lipocalin
NE: Norepinephrine
NF-κB: Nuclear factor-κB
PFA: Paraformaldehyde
Pen/Strep: Penicillin/Streptomycin
PMT: Photomultiplier tube
PBS: Phosphate-buffered saline
PNAs: Platelet-neutrophil aggregates
PRP: Platelet-rich plasma
PPP: Platelet-poor plasma
RMECs: Rat mesenteric endothelial cells
ROI: Region of interest
RT: Room temperature
SLO: Scanning laser ophthalmoscope
SOFA: Sequential Organ Failure Assessment
SSPs: Septic shock patients
SFM: Serum-Free Medium
siRNA^*TRPM7*^: SiRNA against TRPM7
siRNA^*Nontarget*^: SiRNA non-targeting
siRNA: Small interfering RNA
SOCE: Store Operated Calcium Entry
STIM1: Stromal interaction molecule 1
*fli1*:eGFP: Tg(*fli1*:eGFP)^y1^
TRPM7: Transient reception potential melastatin 7
TBIL: Total bilirubin
*trpm7* crispant^*fli1:eGFP*^: *trpm7* crispant in a *fli1:eGFP* genetic background
*trpm7* crispant^*WT*^: *trpm7* Crispant in a WT genetic background
vWF: Von Willebrand factor
WT: Wild type AB
