# Correction: Disseminated intravascular coagulation phenotype is regulated by the TRPM7 channel during sepsis

**DOI:** 10.1186/s40659-023-00433-6

**Published:** 2023-05-11

**Authors:** Ivanka Jiménez-Dinamarca, Yolanda Prado, Pablo Tapia, Sebastian Gatica, Clemens Alt, Charles P. Lin, Cristian Reyes-Martínez, Carmen G. Feijóo, Cristobal Aravena, Alejandra González-Canacer, Simón Correa, Diego Varela, Claudio Cabello-Verrugio, Felipe Simon

**Affiliations:** 1 Laboratory of Integrative Physiopathology, Faculty of Life Sciences, Universidad Andres Bello, Republica 330, 8370186 Santiago, Chile; 2 Millennium Institute On Immunology and Immunotherapy, Santiago, Chile; 3 Unidad de Paciente Crítico Adulto, Hospital Clínico La Florida, Santiago, Chile; 4 Center for Systems Biology and Wellman Center for Photomedicine, Massachusetts General Hospital and Harvard Medical School, Boston, MA USA; 5 Fish Immunology Laboratory, Faculty of Life Sciences, Universidad Andres Bello, Santiago, Chile; 6 Programa de Fisiología Y Biofísica, Facultad de Medicina, Instituto de Ciencias Biomédicas, Universidad de Chile, Santiago, Chile; 7 Millennium Nucleus of Ion Channel-Associated Diseases, Santiago, Chile; 8 Laboratory of Muscle Pathology, Fragility and Aging, Faculty of Life Sciences, Universidad Andres Bello, Republica 330, 8370186 Santiago, Chile; 9 Center for the Development of Nanoscience and Nanotechnology (CEDENNA), Universidad de Santiago de Chile, Santiago, Chile

**Correction: Biological Research (2023) 56:8** **https://doi.org/10.1186/s40659-023-00419-4**

Following publication of the original article [1], the authors identified an error in Figure 4.

The original image in the panel H was inadvertently substituted by a copy of the image showed in panel I (just slightly moved to the left). In the corrected Fig. 4, panel H was changed by the original image. Arrowheads were not changed because they were correctly positioned in the original image. The images showed in H–K are representative of 13 videos for each condition, which analysis is shown in Fig. 4L. All videos are fully available.

**Fig. 4:**
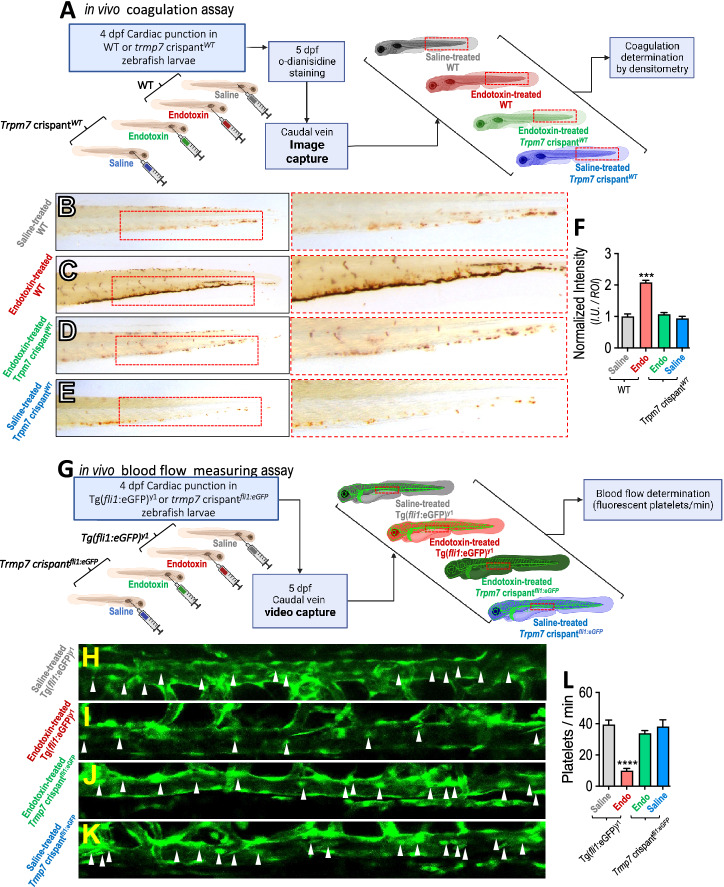
Administration of endotoxin induces coagulation in zebrafish vasculature mediated by TRPM7. (**A**) WT and *trpm7* crispant^*WT*^* zebrafish larvae* were subjected to o-dianisidine staining to evaluate in vivo coagulation. Larvae were injected with 20 nL sterile saline solution (NaCl 0,09%), or endotoxin (LPS (O55:B5 Sigma, USA) 100 ng). Thrombus formation was analyzed 24 h post injection in the caudal vein by o-dianisidine staining. (**B**–**E**) Representative images of saline-injected WT (**B**) endotoxin-injected WT (**C**), endotoxin-injected *trpm7* crispant^*WT*^ (**D**) and saline-treated *trpm7* crispant^*WT*^ conditions (**E**). Doted red box depicts o-dianisidine staining. **F** Quantification of o-dianisidine staining in caudal vein of *Zebrafish larvae* in saline-injected WT (grey bars), endotoxin-injected WT (red bars), endotoxin-injected *trpm7* crispant^*WT*^ (green bars) and saline-treated *trpm7* crispant^*WT*^ (blue bars) conditions. Results of the total pixel intensity (I.U.) in a defined region of interest (ROI), were normalized with the median value of saline condition. Tg(fli1:eGFP)^y1^ and *trpm7* crispant^*fli1:eGFP*^* zebrafish larvae*, having the vasculature and thrombocytes fluorescently green labeled, were subjected to time lapse analysis to evaluate blood flow in vivo coagulation. Blood flow time lapse analysis was determined as the number of platelets observed in 60 s in a section of the caudal vein (doted red box) were performed by time lapse analysis, in saline- and endotoxin-injected conditions (**G**). **H**–**K** Representative images of Tg(fli1:eGFP)^y1^ larvae saline-injected Tg(*fli1*:eGFP)^y1^ (**H**), endotoxin-injected Tg(*fli1*:eGFP)^y1^ (**I**), endotoxin-injected *trpm7* crispant^*fli1:eGFP*^ (**J**), and saline-treated *trpm7* crispant^*fli1:eGFP*^ conditions (**K**). **L** Quantification of blood flow time lapse analysis in a section of the caudal vein of Tg(*fli1*:eGFP)^y1^ *larvae* in saline-injected Tg(*fli1*:eGFP)^y1^ (grey bars), endotoxin-injected Tg(*fli1*:eGFP)^y1^ (red bars), endotoxin-injected *trpm7* crispant^*fli1:eGFP*^ (green bars) and saline-treated *trpm7* crispant^*fli1:eGFP*^ (blue bars) conditions. Statistical differences were assessed by a one-way analysis of variance (ANOVA) (Kruskal–Wallis) followed by Dunn's post hoc test. ***p < 0.001, ****p < 0.0001, compared with the saline-treated WT or Tg(*fli1*:eGFP)^y1^ conditions. Results showed as mean ± SEM

The correct figure is given.

Additional file 7 should be current additional file 9, additional file 8 should be current additional file 7, and additional file 9 should be current additional file 8.

The original article has been corrected.

## References

[1] Jiménez-Dinamarca I, Prado Y, Tapia P, Gatica S, Alt C, Lin CP, Reyes-Martínez C, Feijóo CG, Aravena C, González-Canacer A, Correa S, Varela D, Cabello-Verrugio C, Simon F (2023). Disseminated intravascular coagulation phenotype is regulated by the TRPM7 channel during sepsis. Biol Res.

