# Supplementary material for: Disseminated intravascular coagulation phenotype is regulated by the TRPM7 channel during sepsis
Source: Biol Res. 2023 Mar 3;56:8. doi: 10.1186/s40659-023-00419-4 (PMC9983216; doi:10.1186/s40659-023-00419-4)

# Supplemental information

## Disseminated intravascular coagulation phenotype is regulated by the TRPM7 ion channel during sepsis.

Ivanka Jiménez-Dinamarca[1,‡], Yolanda Prado[1,2,‡], Pablo Tapia[3], Sebastian Gatica[1,2], Clemens Alt[4], Charles Lin[4], Cristian Reyes-Martínez[5], Carmen Gloria Feijóo[5], Cristobal Aravena[1], Alejandra González-Canacer[1], Simón Correa[1], Diego Varela[6,7], Claudio Cabello-Verrugio[2,8,9,*], and Felipe Simon[1,2,7,*]

[1]Laboratory of Integrative Physiopathology, Faculty of Life Science, Universidad Andres Bello, Santiago, Chile.

[2]Millennium Institute on Immunology and Immunotherapy, Santiago, Chile.

[3]Unidad de Paciente Crítico Adulto, Hospital Clínico La Florida, La Florida, Santiago, Chile.

[4]Center for Systems Biology and Wellman Center for Photomedicine, Massachusetts General Hospital and Harvard Medical School, Boston, MA, USA.

[5]Fish Immunology Laboratory, Faculty of Life Science, Universidad Andres Bello, Santiago, Chile.

[6]Programa de Fisiología y Biofísica, Instituto de Ciencias Biomédicas, Facultad de Medicina, Universidad de Chile, Santiago, Chile.

[7]Millennium Nucleus of Ion Channel-Associated Diseases (MiNICAD), Santiago, Chile.

[8]Laboratory of Muscle Pathology, Fragility and Aging, Faculty of Life Science, Universidad Andres Bello, Santiago, Chile.

[9]Center for the Development of Nanoscience and Nanotechnology (CEDENNA), Universidad de Santiago de Chile, Santiago, Chile.

[‡]: These authors contributed equally to this work.

[*]: Corresponding authors

## SUPPLEMENTAL MATERIAL AND METHODS

### Cell Culture

Human umbilical vein endothelial cell (HUVEC)-derived endothelial cell line EA.hy926 was cultured in Dulbecco's Modified Eagle Medium Low (DMEM-Low) with 10% fetal bovine serum (FBS), 4 mM l-glutamine, 1 g/L d-glucose, 100 µg/mL penicillin/streptomycin (Pen/Strep) and 2.5 µg/mL amphotericin. Cells were grown in an incubator at 37 °C in a humidified atmosphere (95% air and 5% $CO_2$)

### Measurement of $[Ca^{2+}]_i$.

ECs were harvested with trypsin/EDTA, washed twice in ice-cold PBS, resuspended and loaded with the $Ca^{2+}$-sensitive cell permeant dye Fluo-4 (5 µM) for 15-30 min at room temperature in the dark. Vehicle- or endotoxin-treated ECs in the absence or presence of carvacrol or 2-APB, or vehicle- or endotoxin-treated ECs transfected with $siRNA^{TRPM7}$ or $siRNA^{Nontarget}$, were analyzed immediately by flow cytometry (FACSCanto, BD Biosciences, San José, CA). Experiments were performed and then intracellular calcium levels were measured using Fluo-4 dye. A minimum of 10,000 cells/sample were analyzed. Cellular dye intensity analysis was performed using FACSDiva software v4.1.1 (BD Biosciences).

### Plasma membrane TRPM7 determination

Flow cytometry analysis was performed to determine expression changes of TRPM7 using a TRPM7 monoclonal antibody that recognize an extracellular epitope (ECM Biosciences, USA) in non-permeabilized conditions, coupled to suitable secondary antibodies conjugated to fluorophores (ThermoFisher, USA). The labeled cells were then analyzed immediately by flow cytometry (BD FACS Fortessa, BD Biosciences, USA). Color compensation matrices were calculated for each staining combination within each experiment using single-stained antibody. In all analyses, doublets and clusters were eliminated. A minimum of 10,000 events were analyzed.

### Quantitative RT-PCR

QPCR experiments were performed to measure the TRPM7, TRPM6 and TRPM2 mRNA expression in endothelial cells infected with $AdV^{shTRPM7}$, $AdV^{CTRL}$, or AdV vehicle (Non-infected). Total RNA was extracted with Trizol according to the manufacturer's protocol (Invitrogen, Carlsbad, CA). DNAse I-treated RNA was used for reverse transcription using the Super Script II Kit (Invitrogen, Carlsbad, CA). Equal amounts of RNA were used as templates in each reaction. QPCR was performed using the SYBR Green PCR Master Mix (AB Applied Biosystems, Foster City, CA). Assays were run using a Rotor-gene system (Corbet Research) instrument. Data are presented as relative mRNA levels of the gene of interest normalized to relative levels of 28S mRNA.

## SUPPLEMENTAL TABLE LEGENDS

**Supplemental Table S1. Guide RNAs.**

**Supplemental Table S2. Demographic characteristics and clinical data of SSP and healthy volunteers**

**Supplemental Table S3. Characteristics of septic shock patients (SSP)**

**Supplemental Table S4. Cardiorespiratory measurements in endotoxic and control rats.** Percent change of variables 90 min after *i.p.* injection of endotoxin (20 mg/kg) or sterile saline solution (control). Values are expressed as mean ± SEM.

**Supplemental Table S5. Cytokines measurements in endotoxic and control rats.** Fold of change of cytokines 90 min after i.p. injection of endotoxin (20 mg/kg) or sterile saline solution (control). Values are expressed as mean ± SEM.

## SUPPLEMENTAL FIGURE LEGENDS

**Supplemental Figure S1. TRPM7 crispant phenotype. A**. Scheme of the *trpm7* genomic sequence, showing the exons (blue rectangle) to which gRNA were target. (**B, C**) Lateral and dorsal view of a 3 dpf control larva. (**D, E**) Lateral and dorsal view of a 3 dpf *trpm7* crispant larva.

**Supplemental Figure S2. Endotoxin-induced intracellular calcium is regulated by TRPM7 ion channel.** (**A**) ECs were preincubated for 1 h in the presence or absence TRPM7 inhibitors carvacrol and 2-APB, and then exposed to endotoxin (10 μg/mL) for 24 h, after which, relative $[Ca^{2+}]_i$ was measured. Results are expressed as normalized Fluo-4 fluorescence compared to control. Statistical differences were assessed by two-way analysis of variance (ANOVA) followed by Tukey's *post hoc* test. ***: $p < 0.001$, against vehicle condition. ($N = 7$). (**B**) ECs were transfected with a siRNA$^{TRPM7}$, siRNA$^{Nontarget}$ or non-transfected and TRPM7 mRNA expression was measured. Results are expressed as normalized TRPM7 mRNA expression compared to non-transfected condition. Statistical differences were assessed by one-way analysis of variance (ANOVA) followed by Dunns's *post hoc* test. ****: $p < 0.0001$, against vehicle condition. ($N = 7$). (**C**) ECs were transfected with a siRNA$^{\mathrm{TRPM7}}$, siRNA$^{\mathrm{Non\text{-}target}}$ or non-transfected, after which, relative $[Ca^{2+}]_i$ was measured. Results are expressed as normalized Fluo-4 fluorescence compared to control. Statistical differences were assessed by two-way analysis of variance (ANOVA) followed by Tukey's *post hoc* test. ***: $p < 0.001$, against vehicle condition. ($N = 7$). Results are expressed as the mean ± SEM. (**D-E**) TRPM7 detection in plasma membrane of ECs. Vehicle- and endotoxin-treated ECs exposed to carvacrol or 2-APB (**D**) or transfected with a siRNA against TRPM7 (siRNA$^{TRPM7}$) or transfected with a siRNA control (siRNA$^{Nontarget}$) (**E**). Statistical differences were assessed by a two-way analysis of variance (ANOVA) followed by Tukey *post hoc* test. **$p < 0.01$, compared with the vehicle-treated condition in the saline- or non-transfected-condition. Results showed as mean ± SEM.

**Supplemental Figure S3. Administration of endotoxin induces coagulation in zebrafish vasculature.** (**A**) WT *zebrafish larvae* were subjected to o-dianisidine staining to evaluate *in vivo* coagulation. Larvae were injected with 20 nL sterile saline solution (NaCl 0,09%), or endotoxin (LPS (O55:B5 Sigma, USA) 100 ng) in

the presence or absence FTY-720 (Tocris, USA) by immersion (0.5 ng/μL) for 24 h after and during endotoxin treatment. Thrombus formation was analyzed 24 h post injection in the caudal vein by o-dianisidine staining. (**B-E**) Representative images of zebrafish saline-injected (**B**) endotoxin-injected (**C**), endotoxin-injected / FTY-treated (**D**) and FTY-treated (**E**). Doted red box depicts o-dianisidine staining. (**F**) Quantification of o-dianisidine staining in caudal vein of *Zebrafish larvae* in saline-injected condition (grey circles), endotoxin-injected (red circles), endotoxin-injected / FTY treated (green circles) and FTY-treated (blue circles). Results of the total pixel intensity (I.U.) in a defined region of interest (ROI), were normalized with the median value of saline condition. Tg(fli1:eGFP)$^{y1}$ *larvae*, having the vasculature and thrombocytes fluorescently green labeled, were subjected to time lapse analysis to evaluate blood flow *in vivo* coagulation. Blood flow time lapse analysis was determined as the number of platelets observed in 60 seconds in a section of the caudal vein (doted red box) were performed by time lapse analysis, in saline-injected, endotoxin-injected, endotoxin-injected / FTY-treated, and FTY-treated conditions (**G**). (**H-K**). Representative images of Tg(fli1:eGFP)$^{y1}$ larvae saline-injected (**H**), endotoxin-injected (**I**), endotoxin-injected / FTY-treated (**J**), and FTY-treated conditions (**K**). (**L**) Quantification of blood flow time lapse analysis in a section of the caudal vein of Tg(fli1:eGFP)$^{y1}$ *larvae* in saline-injected condition (grey circles), endotoxin-injected (red circles), endotoxin-injected / FTY treated (green circles) and FTY-treated (blue circles). Statistical differences were assessed by a one-way analysis of variance (ANOVA) (Kruskal–Wallis) followed by Dunn's *post hoc* test. ***$p < 0.001$, ****$p < 0.0001$, compared with the saline-treated WT or Tg(*fli1*:eGFP)$^{y1}$ conditions. Results showed as mean ± SEM.

**Supplemental Figure S4. Endotoxin-induced vWF, ICAM-1 and P-Sel mRNA expression is regulated by TRPM7 ion channel.** ECs were preincubated for 1 h in the presence or absence TRPM7 inhibitors carvacrol and 2-APB, and then exposed to endotoxin (10 μg/mL) for 24 h, after which, mRNA expression of vWF (**A**), ICAM-1 (**B**) and P-Sel (**C**). Results are expressed as normalized to vehicle condition. Statistical differences were assessed by two-way analysis of variance (ANOVA) followed by Tukey's *post hoc* test. ****: $p < 0.0001$, against vehicle condition. ($N = 6$). ECs were transfected with a siRNA$^{TRPM7}$, siRNA$^{Non\text{-}target}$ or non-transfected and then exposed to endotoxin (10 μg/mL) for 24 h, after which, mRNA expression of vWF (**D**), ICAM-1 (**E**) and P-Sel (**F**). Results are expressed as normalized to vehicle condition. Statistical differences were assessed by

two-way analysis of variance (ANOVA) followed by Tukey's *post hoc* test. ****: $p < 0.0001$, against vehicle condition. ($N = 6$). Results are expressed as the mean ± SEM

**Supplemental Figure S5. In vitro specificity and efficiency of AdV$^{shTRPM7}$ infection to suppress TRPM7 expression**. (**A**) Normalized TRPM7 protein levels expressed relative to non-infected condition (N = 3). (**B**) QPCR for TRPM7, TRPM6 and TRPM2 in cells infected with AdV$^{shTRPM7}$, AdV$^{CTRL}$ (used as a control) and non-infected. (**C**) QPCR for TRPM7 in cells infected with AdV$^{shTRPM7}$, AdV$^{CTRL}$ (used as a control) and non-infected at 1 x $10^7$, 1 x $10^8$ and 1 x $10^9$ dilutions. ***, $p<0.001$. Statistical differences were assessed by a one-way analysis of variance (ANOVA) (Kruskal–Wallis) followed by Dunn's post hoc test. Results are expressed as the mean ± SEM.

**Supplemental Figure S6. In vivo specificity and efficiency of AdV $^{shTRPM7}$ infection to suppress TRPM7 expression**. (**A**) Normalized TRPM7 protein levels expressed relative to non-infected condition (N = 3). (**B**) QPCR for TRPM7, TRPM6 and TRPM2 in cells infected with AdV$^{shTRPM7}$, AdV$^{CTRL}$ (used as a control) and non-infected. ***, $p<0.001$. Statistical differences were assessed by a one-way analysis of variance (ANOVA) (Kruskal–Wallis) followed by Dunn's post hoc test. Results are expressed as the mean ± SEM.

**Supplemental Figure S7. AdV $^{shTRPM7}$ infection did not induce a systemic interferon response in rats**. (**A**) Interferon-β (INF-β) plasma levels were measured in blood from rats infected with AdV$^{shTRPM7}$, AdV$^{CTRL}$ (used as a control) and non-infected. Rats infected with an INF-β inducer were used as a positive control (PC). (**B**) Representative images from western blot experiments performed for detection of PKR protein expression in primary rat mesenteric endothelial cells (RMEC) extracted from rats infected with AdV$^{shTRPM7}$, AdV$^{CTRL}$ (used as a control) and non-infected. Rats infected with an INF-β inducer was used as a positive control (PC) (**B, upper panel)**. Densitometric analyses of the experiments shown in A (**B, lower panel)**. PKR protein levels were normalized against tubulin and expressed relative to non-infected condition. ***, $p<0.001$. Statistical differences were assessed by a one-way analysis of variance (ANOVA) (Kruskal–Wallis) followed by Dunn's post hoc test. (N = 3). Results are expressed as the mean ± SEM. (**C**) Survival (Kaplan–Meier) curves comparing saline-

treated (grey dashed line) (N = 16), AdV$^{CTRL}$-injected + saline-treated (red dashed line) (N = 16), AdV$^{shTRPM7}$-injected + saline-treated (green dashed line) (N = 16), AdV vehicle-injected + saline-treated (blue dashed line) (N = 16) rats. NS: non-significant (log-rank (Mantel–Cox) test) when comparing all condition against saline-treated conditions. ns: non-significant (Gehan-Breslow-Wilcoxon test) when comparing all condition against saline-treated conditions.

**Supplemental Figure S8. AdV$^{shTRPM7}$ infected endotoxemic rats are protected from creatinine and urea increase induced by endotoxemia.** Creatinine (**A**) and urea (**B**) were measured in saline and endotoxic rats infected with AdV$^{shTRPM7}$ or AdV$^{CTRL}$. Statistical differences were assessed by one-way analysis of variance (ANOVA) followed by Dunns's *post hoc* test. *: $p < 0.05$, ***: $p < 0.001$ compared with Saline/AdV$^{CTRL}$ condition. (N=16 per group). Results are expressed as the mean ± SEM.

**Supplemental Figure S9. Separation of CMEC and CPEC from SSP and HV.** CMEC were magnetic bead-based immunoseparated from HV, and SSP blood samples and TRPM7 expression analyses were performed.

**SUPPLEMENTAL MOVIE LEGENDS**

**Supplemental Movie S1.** Representative time lapse of retinal circulation from endotoxic mouse (LPS 3 mg/kg i.p. ) at 3 h showing neutrophil-endothelial interaction (NEI).

**Supplemental Movie S2.** Representative time lapse of retinal circulation from endotoxic mouse (LPS 3 mg/kg i.p. ) at 12 h showing neutrophil-endothelial interaction (NEI).

**Supplemental Movie S3.** Representative time lapse of retinal circulation from endotoxic mouse (LPS 3 mg/kg i.p. ) at 3 h showing neutrophil-endothelial interaction (NEI) in the presence of Carvacrol (80 mg/kg i.p., 1 h post-endotoxemia).

**Supplemental Movie S4.** Representative time lapse of retinal circulation from endotoxic mouse (LPS 3 mg/kg i.p. ) at 12 h showing neutrophil-endothelial interaction (NEI) in the presence of Carvacrol (80 mg/kg i.p., 1 h post-endotoxemia).

**Supplemental Movie S5.** Representative time lapse of Tg(*fli1*:eGFP)$^{y1}$ 4 dpf larvae microinjected in the heart with 20 nL of saline buffer (NaCl 0,9%)

**Supplemental Movie S6.** Representative time lapse of Tg(*fli1*:eGFP)$^{y1}$ 4 dpf larvae microinjected in the heart with 20 nL of endotoxin (LPS 100 ng).

**Supplemental Movie S7.** Representative time lapse of *trmp7* crispant$^{fli1:eGFP}$ 4 dpf larvae microinjected in the heart with 20 nL of endotoxin (LPS 100 ng).

**Supplemental Movie S8.** Representative time lapse of *trmp7* crispant$^{fli1:eGFP}$ 4 dpf larvae microinjected in the heart with 20 nL of saline buffer (NaCl 0,9%)

**Supplemental Movie S9.** Representative time lapse of Tg(fli1:eGFP)$^{y1}$ 4 dpf larvae microinjected in the heart with 20 nL of saline buffer (NaCl 0,9%)

**Supplemental Movie S10.** Representative time lapse of Tg(fli1:eGFP)$^{y1}$ 4 dpf larvae microinjected in the heart with 20 nL of endotoxin (LPS 100 ng).

**Supplemental Movie S11.** Representative time lapse of Tg(fli1:eGFP)$^{y1}$ 4 dpf larvae microinjected in the heart with 20 nL of endotoxin (LPS 100 ng) and treated by immersion with the TRPM7 inhibitor FTY-720 (0.5 ng/μL).

**Supplemental Movie S12.** Representative time lapse of Tg(fli1:eGFP)$^{y1}$ 4 dpf larvae microinjected in the heart with 20 nL of saline buffer (NaCl 0,9%) and treated by immersion with the TRPM7 inhibitor FTY-720 (0.5 ng/μL).

# Supplemental Table S1

**Supplemental Table S1. Guide RNAs.**

| Name | Target sequence (5' to 3') | gRNA primer sequence (5' to 3') |
|---|---|---|
| gRNA 1 | TCTTTGGCCAGAGTCCACGACGG | TAATACGACTCACTATATCTTTGGCCAGAGTCCACGAGTTTTAGAGCTAGAA |
| gRNA 2 | GGGGTCATCAACTTCCAGGGCGG | TAATACGACTCACTATAGGGGTCATCAACTTCCAGGGGTTTTAGAGCTAGAA |
| gRNA 3 | CTGGCAGACCATCTACAGGGAGG | TAATACGACTCACTATACTGGCAGACCATCTACAGGGGTTTTAGAGCTAGAA |
| gRNA 4 | AGTGCCGACCAACCTTCTGGAGG | TAATACGACTCACTATAAGTGCCGACCAACCTTCTGGGTTTTAGAGCTAGAA |

# Supplemental Table S2

**Supplemental Table S2. Demographic characteristics and clinical data of SSP and healthy volunteers**

| Variable | Healthy Volunteers (n=25) | UCI SSP (n=22) |
|---|---|---|
| **Mortality at 30 days, %** | 0 | 54,5 |
| **APACHE II, median (IQR)** | ND | 22.6 (19.6-25,5) |
| **SOFA, median (IQR)** | ND | 12,3 (9,2-13,5) |
| **Age (ys), median (IQR)** | 62 (44-69) | 65 (54-75) |
| **BMI, median (IQR)** | 25.6 (21.7-28.4) | 26.3 (24.2-29.8) |
| **Male sex, (% of male)** | 56 | 54 |
| **Time of sampling (h)*, median (IQR)** | NA | 47 (45-72) |
| **CRP (mg/dl), median (IQR)** | ND | 31,6 ( 25.4-31.9) |
| **Resuscitation Fluid (Liter),median (IQR)** | NA | 6.0 (5.2 -7.1) |
| **Blood lactate level (mmol/L)median (IQR)** | ND | 8.9 (7.1-12.6) |
| **Norepinephrine (ug/kg/min) median (IQR)** | NA | 0.30 (0.23-0.56) |
| **Renal replacement therapy(%)** | 0 | 26,2 |
| **Corticosteroids, %** | NA | 79 |
| **Mechanical ventilation, %** | 0 | 100 |

*Definition of abbreviations:*
BMI, body mass index;
CRP, C-reactive protein;
APACHE-II, Acute Physiology and Chronic Health Evaluation II score;
SOFA, Sepsis-related Organ Failure Assessment,
IQR: interquartile range (expressed as percentile 25th, percentile 75th),
ND: non-determined,
NA: non-applicable
* The sample for analysis was collected always prior to connection to a renal replacement therapy.

# Supplemental Table S3

**Supplemental Table S3. Characteristics of septic shock patients (SSP)**

| SSP | Diagnosis | Etiology |
|---|---|---|
| **1** | Peritonitis | Polymicrobial infections |
| **2** | Urosepsis | E coli |
| **3** | Pneumonia | S. pneumoniae |
| **4** | Pneumonia | S. aureus |
| **5** | Urosepsis | E. coli |
| **6** | Pneumonia | S. aureus |
| **7** | Meningitis | S. pneumoniae |
| **8** | Cholangitis | K. pneumoniae |
| **9** | Cholangitis | C. freundii |
| **10** | Necrotising fasciitis | S. aureus |
| **11** | Pneumonia | S. pneumoniae |
| **12** | Spondylodiscitis | S. aureus |
| **13** | Urosepsis | K. pneumoniae |
| **14** | Pneumonia | S. pneumoniae |
| **15** | Cholangitis | E. coli |
| **16** | Meningitis | S. pneumoniae |
| **17** | Pneumonia | S. pneumoniae |
| **18** | Peritonitis | Polymicrobial |
| **19** | Bacteremia | E coli |
| **20** | Necrotising fasciitis | S. pyogenes |
| **21** | Liver abscess | K. pneumoniae |
| **22** | Severe colitis | C. difficile |

*Definition of abbreviations:*
C. difficile, Clostridium difficile;
S. pneumoniae, Streptococcus pneumoniae;
E. coli, Escherichia coli;
S. aureus, Staphylococcus aureus;
S. piogenes, Streptococcus piogenes
C. freundii , Citrobacter freudii;
K. pneumoniae , Klebsiella pneumoniae;

# Supplemental Table S4

**Supplemental Table S4. Cardiorespiratory measurements in endotoxic and control rats.** Percent change of variables 90 min after *i.p.* injection of endotoxin (20 mg/kg) or sterile saline solution (control). Values are expressed as mean ± SEM.

| Variable | Δ vs. control (%) | *p*-value |
|---|---|---|
| Ps | – 23.7 ± 3.2 | $p < 0.05$ |
| $f_H$ | + 18.3 ± 2.1 | $p < 0.05$ |

Ps = systolic blood pressure; $f_H$ = heart rate.

# Supplemental Table S5

**Supplemental Table S5. Cytokines measurements in endotoxic and control rats.**
Fold of change of cytokines 90 min after i.p. injection of endotoxin (20 mg/kg) or sterile saline solution (control). Values are expressed as mean ± SEM.

| Variable | Δ vs. control (fold of change) | *p*-value |
|---|---|---|
| TNF-α | + 2.3 ± 0.21 | $p < 0.05$ |
| IL-1β | + 3.3 ± 0.18 | $p < 0.05$ |
| IL-6 | +2.1 ± 0.5 | $p < 0.05$ |

## Supplemental Figure S1

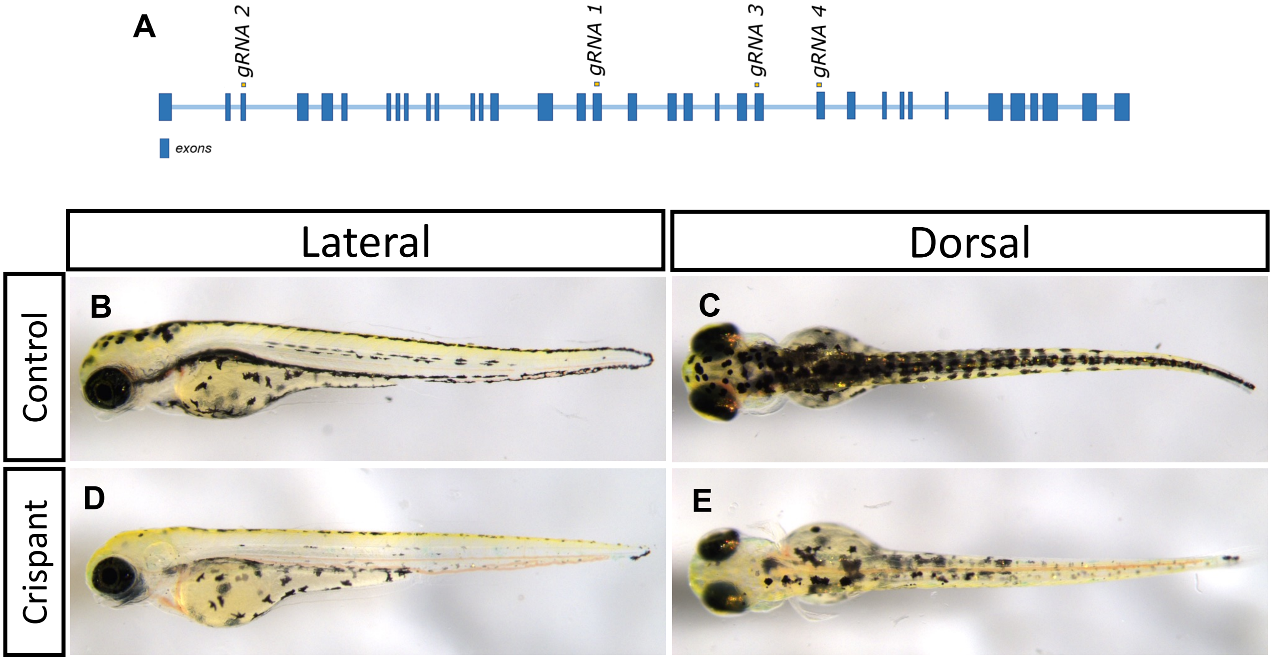

# Supplemental Figure S2

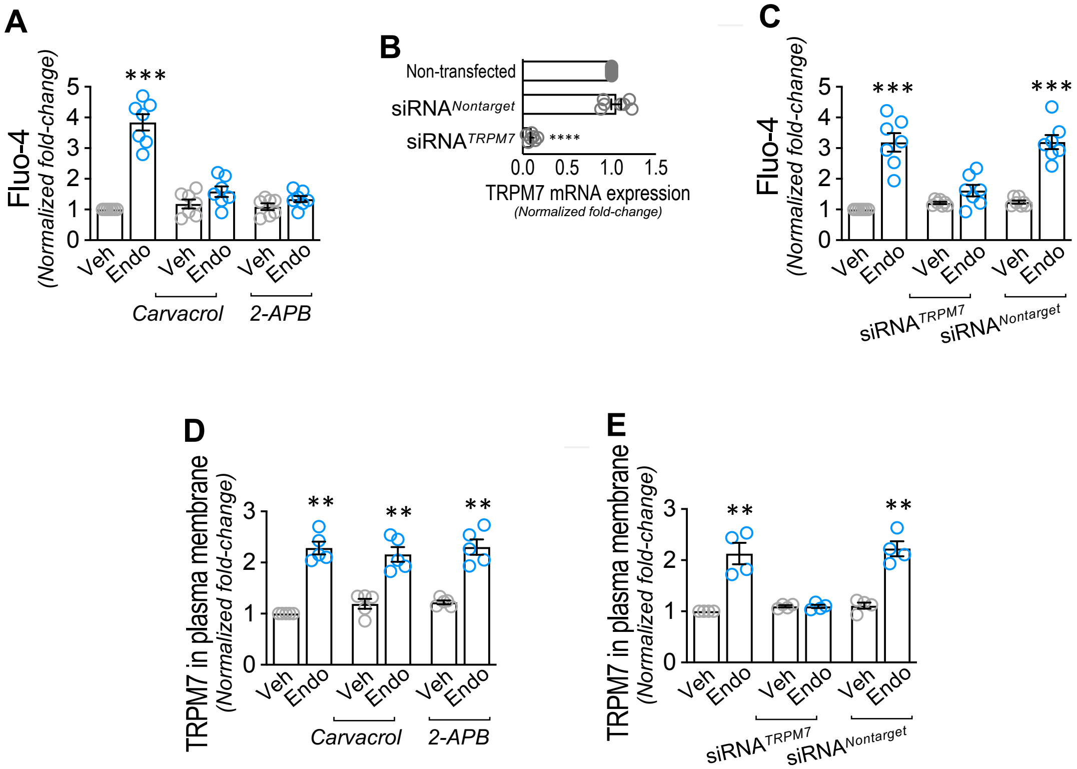

# Supplemental Figure S3

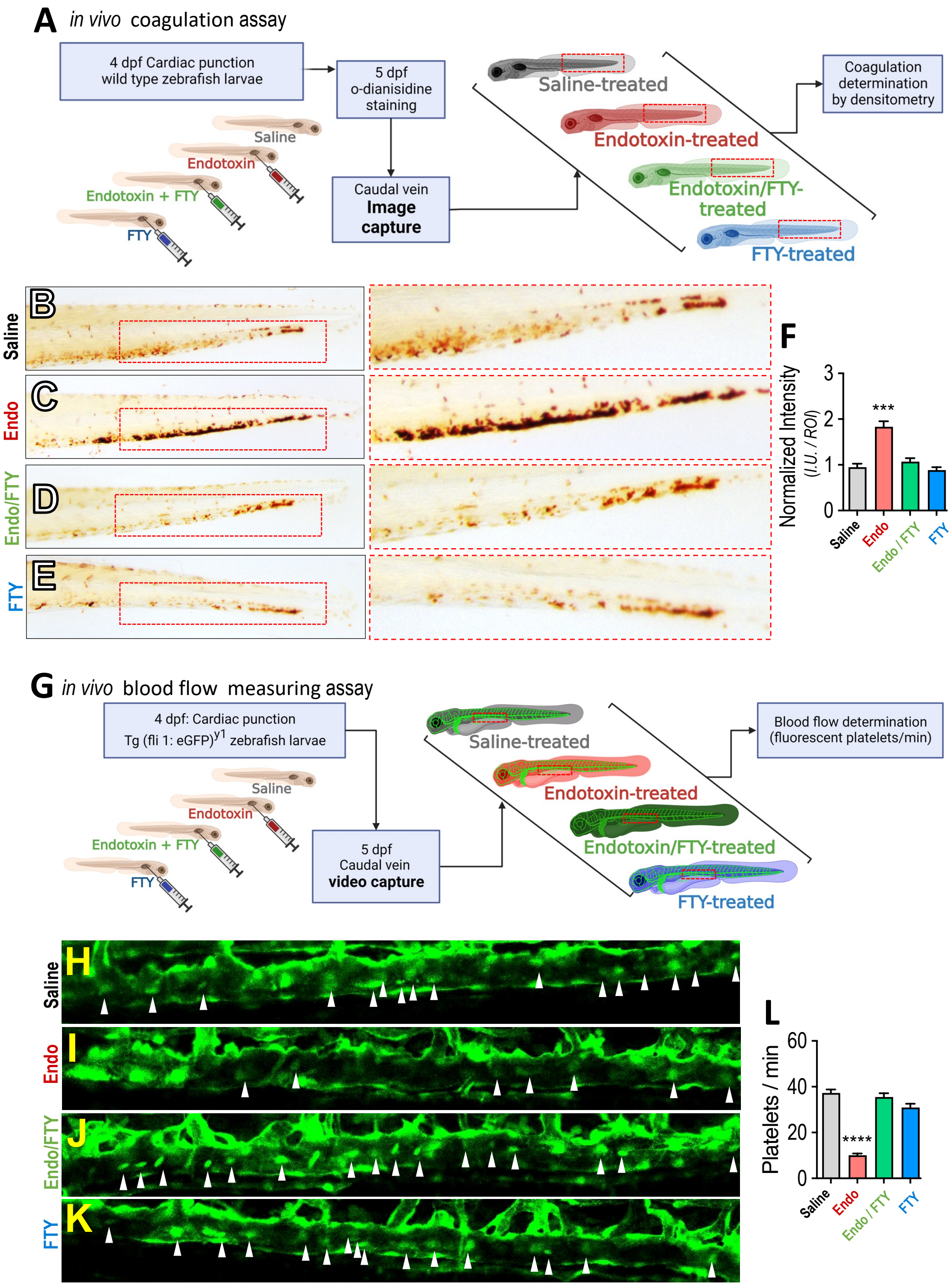

# Supplemental Figure S4

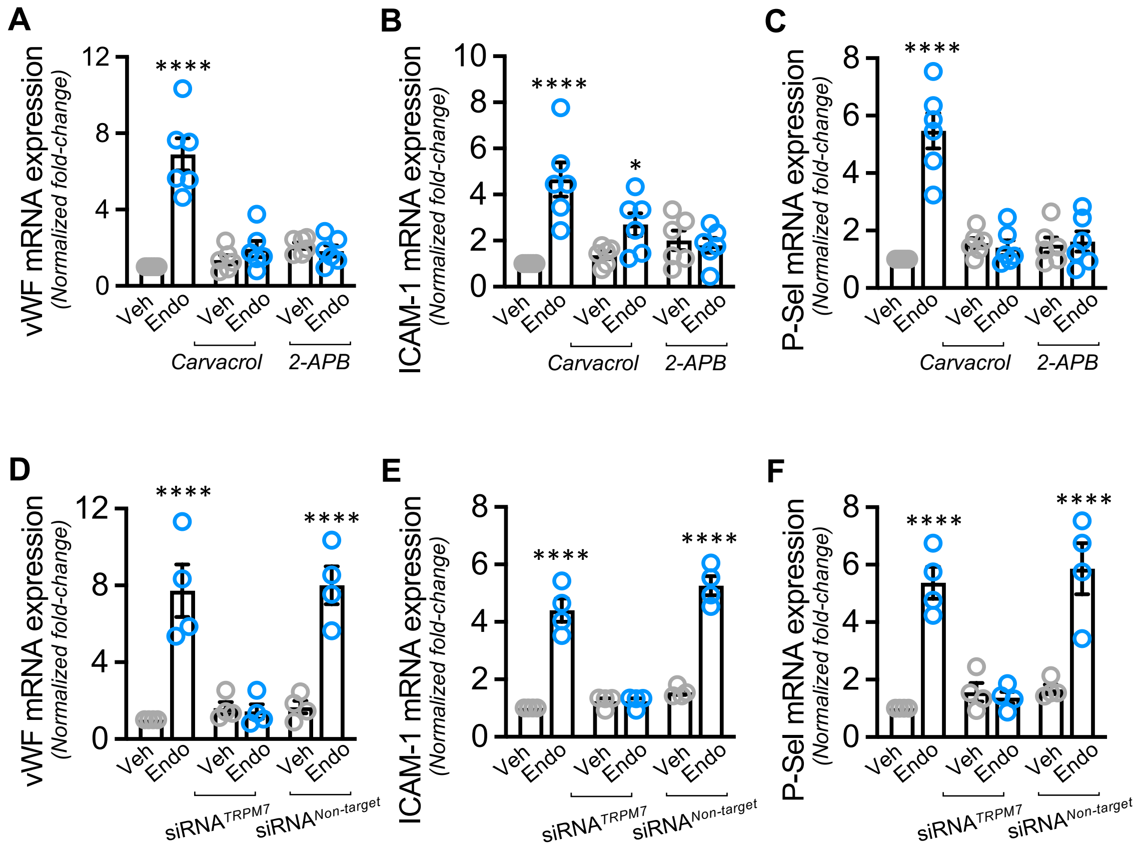

# Supplemental Figure S5

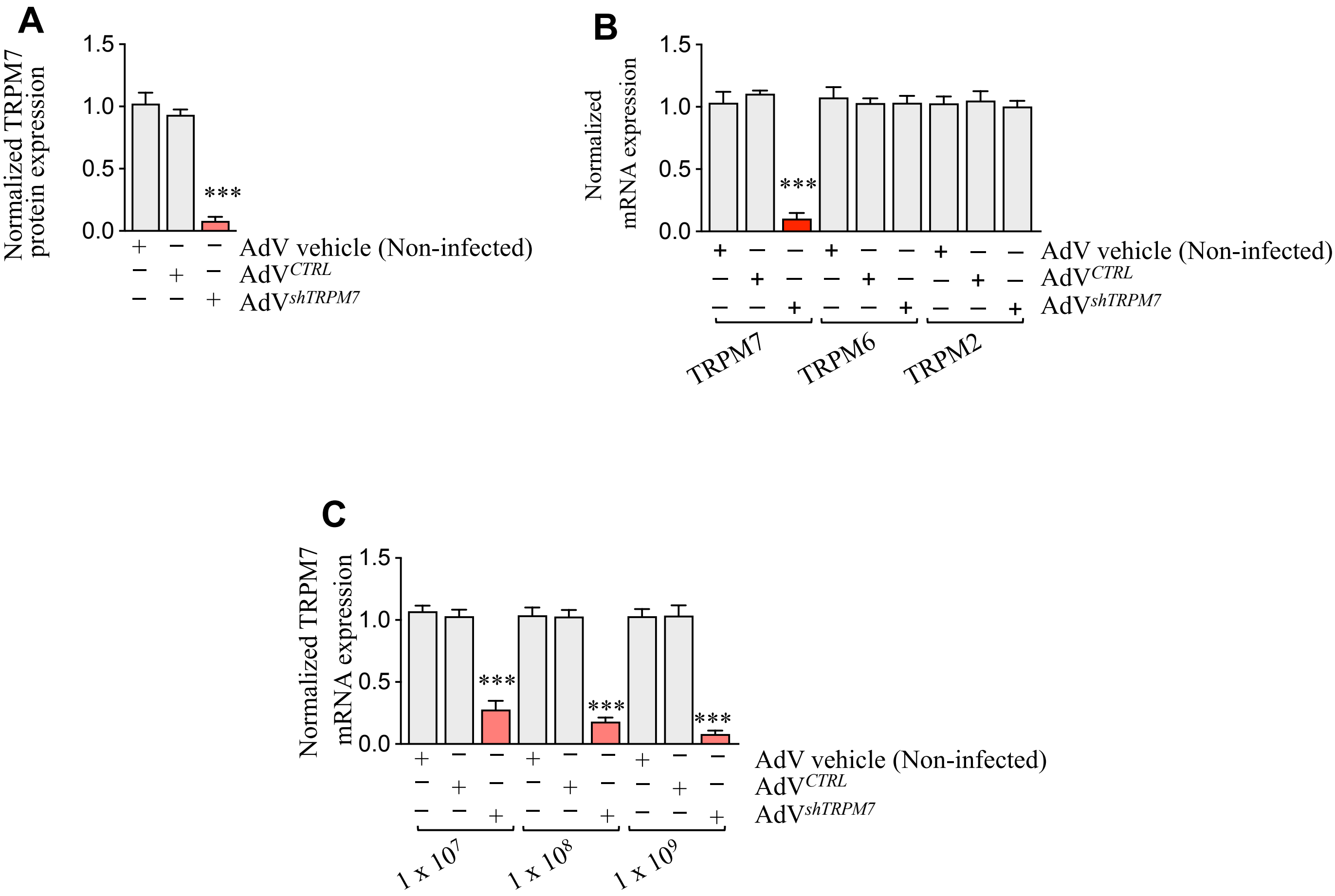

# Supplemental Figure S6

A

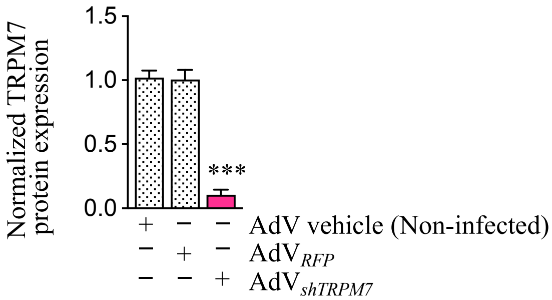


B

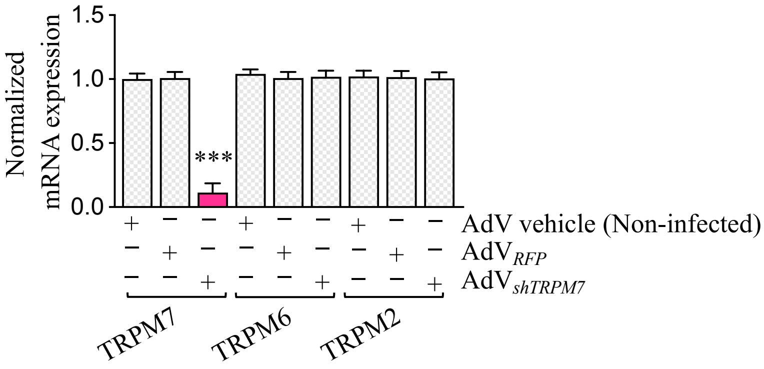

# Supplemental Figure S7

A

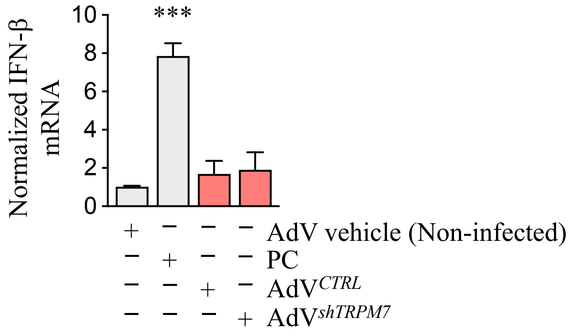


B

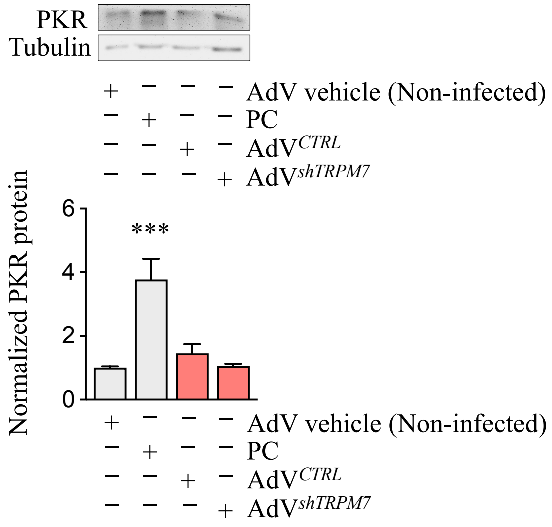


C

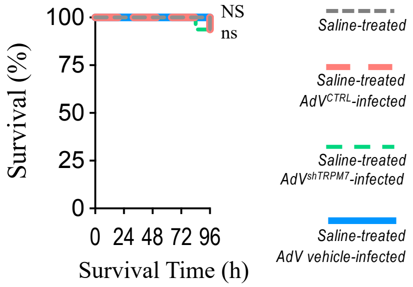

# Supplemental Figure S8

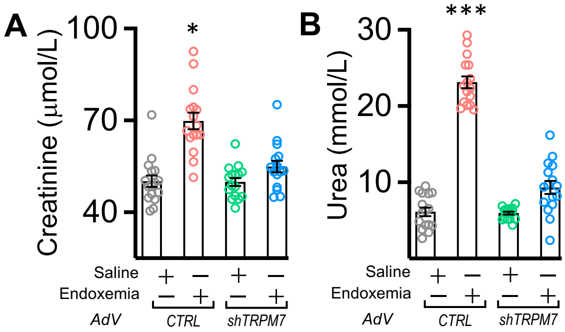

# Supplemental Figure S9

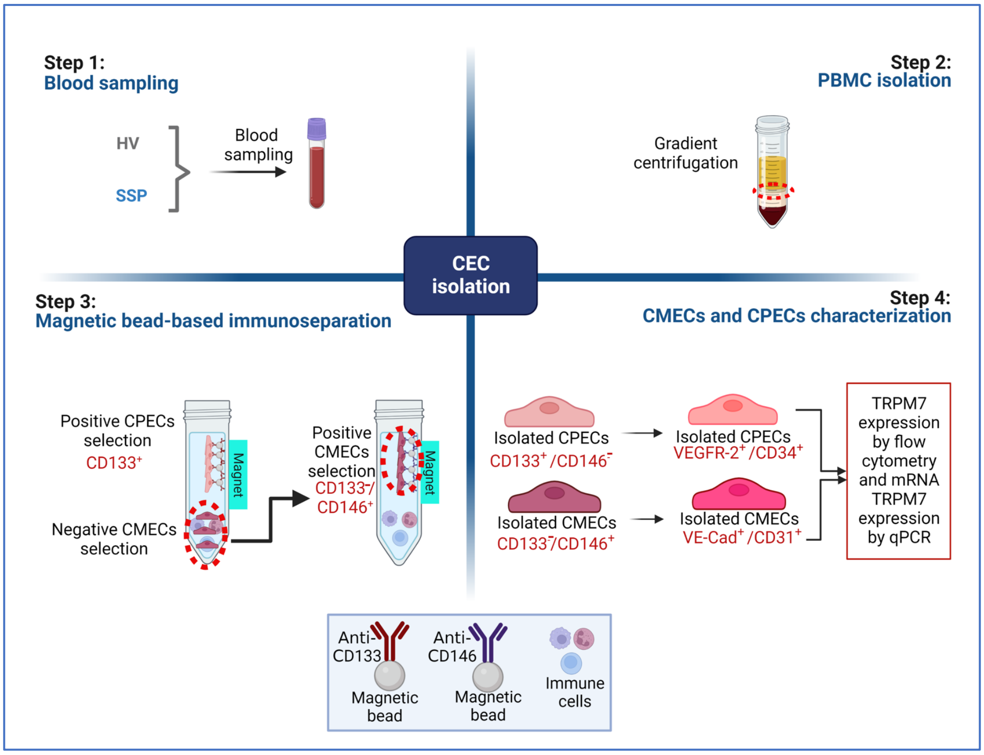


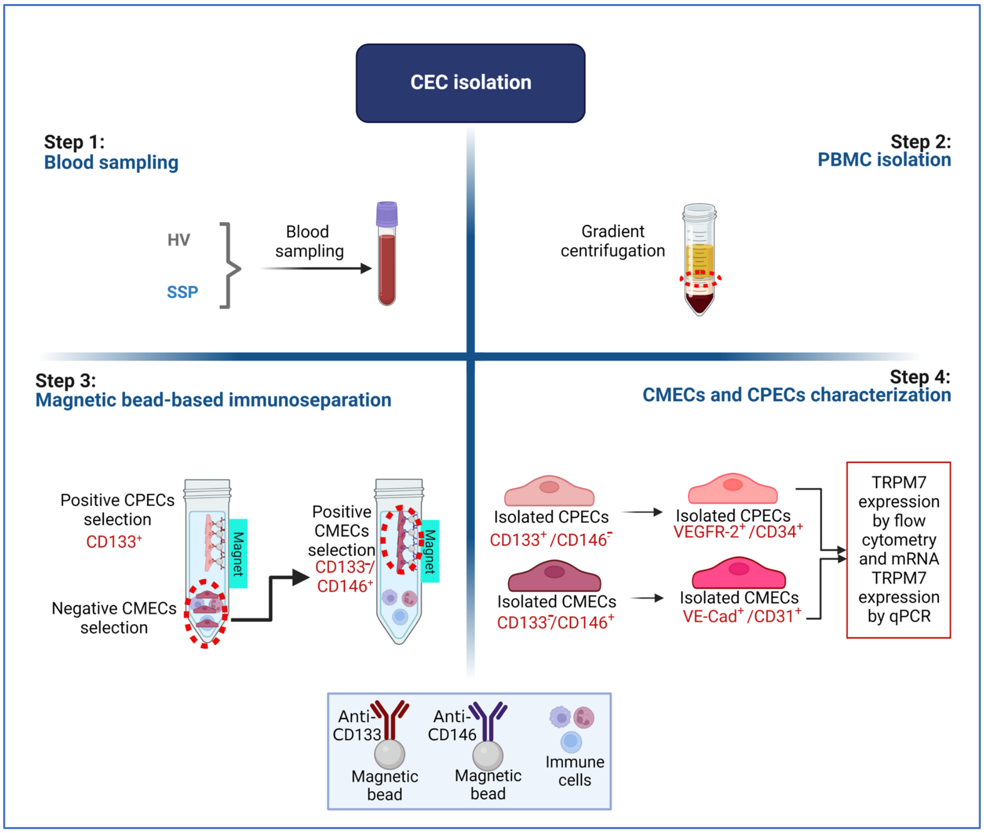

Supplement: Supplementary file 1 — Additional file 1. Supplemental information. [file 40659_2023_419_MOESM1_ESM.pdf]
